# Exciton Diffusion in Highly-Ordered One Dimensional Conjugated Polymers: Effects of Back-Bone Torsion, Electronic Symmetry, Phonons and Annihilation

**DOI:** 10.1021/acs.jpclett.1c00193

**Published:** 2021-04-08

**Authors:** Raj Pandya, Antonios M. Alvertis, Qifei Gu, Jooyoung Sung, Laurent Legrand, David Kréher, Thierry Barisien, Alex W. Chin, Christoph Schnedermann, Akshay Rao

**Affiliations:** † Cavendish Laboratory, University of Cambridge, J.J. Thomson Avenue, CB3 0HE, Cambridge, United Kingdom; ‡ Sorbonne Université, CNRS, Institut des NanoSciences de Paris, INSP, 4 place Jussieu, F-75005 Paris, France; § Sorbonne Université, CNRS, Institut Parisien de Chimie Moléculaire (IPCM) UMR 8232, Chimie des Polymères, 4 Place Jussieu, 75005 Paris, France

## Abstract

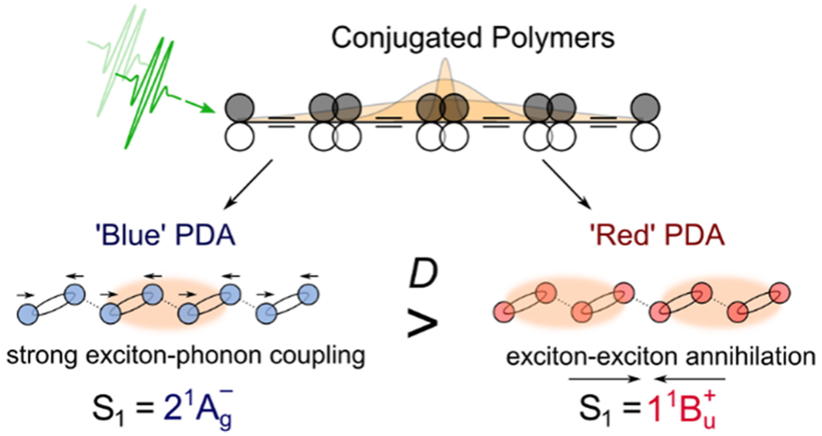

Many optoelectronic devices based on organic materials require rapid and long-range singlet exciton transport. Key factors controlling exciton transport include material structure, exciton–phonon coupling and electronic state symmetry. Here, we employ femtosecond transient absorption microscopy to study the influence of these parameters on exciton transport in one-dimensional conjugated polymers. We find that excitons with 2^1^A_g_^–^ symmetry and a planar backbone exhibit a significantly higher diffusion coefficient (34 ± 10 cm^2^ s^–1^) compared to excitons with 1^1^B_u_^+^ symmetry (7 ± 6 cm^2^ s^–1^) with a twisted backbone. We also find that exciton transport in the 2^1^A_g_^–^ state occurs without exciton–exciton annihilation. Both 2^1^A_g_^–^ and 1^1^B_u_^+^ states are found to exhibit subdiffusive behavior. Ab initio *GW*-BSE calculations reveal that this is due to the comparable strengths of the exciton–phonon interaction and exciton coupling. Our results demonstrate the link between electronic state symmetry, backbone torsion and phonons in exciton transport in π-conjugated polymers.

## Introduction

Organic optoelectronic devices ranging from light-emitting diodes to photovoltaic cells and transistors^1−3^ are frequently based on linear polymers with a *C*_2h_ point group symmetry. Here, the electronic ground state (S_0_) exhibits 1^1^A_g_^+^ symmetry. Depending on the polymer conjugation length and backbone geometry the first (S_1_) and second (S_2_) excited electronic states are either of 2^1^A_g_^–^ or 1^1^B_u_^+^ symmetry.^4,5^ Irrespective of the exact state ordering, photoexcitations in conjugated polymers rapidly form excitons in the lowest energy excited S_1_ state. When the S_1_ state is of 2^1^A_g_^–^ symmetry the materials are typically nonfluorescent, the S_1_ state supports some triplet-pair character ^1^(TT)^6−8^ and frequently shows a short electronic lifetime. On-the-other hand polymers with an S_1_ state of 1^1^B_u_^+^ character are often luminescent with long electronic lifetimes.^9,10^

Despite our considerable understanding of the electronic structure of conjugated polymers,^3,4,11−15^ the impact of the electronic symmetry of the S_1_ state on exciton diffusion behavior remains largely unexplored. In other words, it is not known whether a 2^1^A_g_^–^ or 1^1^B_u_^+^ symmetry state would provide better exciton transport properties. At first glance it might appear that the longer lifetimes of 1^1^B_u_^+^ states would enable longer exciton diffusion lengths. But the significantly altered electronic structure and many-body character of 2^1^A_g_^–^ states may provide access to unique diffusion properties. The experimental study of exciton diffusion in molecular systems is challenging due to the difficulties associated with measuring ultrafast, nanoscale exciton diffusion properties in systems that exhibit sub-100 ps electronic lifetimes coupled with sub-100 nm exciton diffusion lengths.^12,13^ Critically challenges associated with systematically tuning polymer electronic structure without introducing detrimental effects such as interchain disorder, cross-linking, etc. have until now prevented a systematic investigation of electronic symmetry on exciton diffusion.

## Results

**Figure 1:**
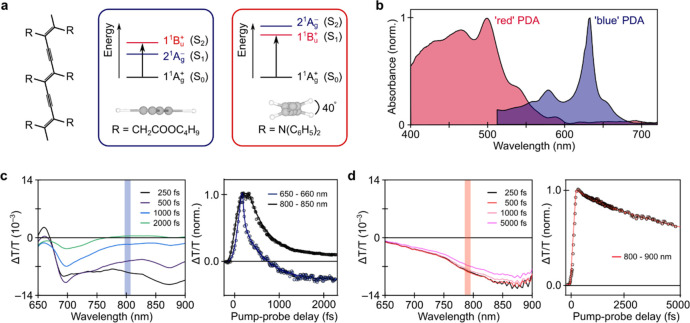
Structural and optical characterization of “blue” and “red” PDA. a. Chemical structure and packing side-chains of “blue” (left) and “red” (right) PDA. In “blue” PDA the backbone is planar, whereas in “red” PDA there is a 40° twist angle between adjacent monomer units in the chain. b. Absorption spectra of “blue” and “red” PDA. c–d. Selected spectral slices of pump–probe spectra of “blue” (c) and “red” PDA (d). The photoinduced absorption bands of the 2^1^A_g_^–^ and 1^1^B_u_^+^ states are marked. Shaded regions indicate probe wavelengths used in transient absorption microscopy experiments. The right panels show kinetics and associated exponential decay fits at indicated probe wavelengths.

Here, we overcome these problems by using femtosecond transient absorption spectroscopy and microscopy^16−19^ to measure exciton transport in different topochemically (i.e., near defect-free as compared to solution processed materials) polymerized polydiacetylene (PDA) chains. PDAs offer an ideal platform to study the effect of electronic properties on exciton transport for several key reasons. Structurally, PDAs consist of extended (2–5 μm) one-dimensional polymer chains that are distinguished by the degree of backbone torsion. Perfectly planar polymer chains are typically referred to as “blue” PDA, while polymer chains with a defined backbone torsional angle (40°) are known as “red” PDA. We note that although the “blue” PDA actually has redder absorption peaks than the “red” PDA, the naming convention is historical and based on the color of the crystals.^20^ In this study the chains are dilutely (polymer content 10^–4^–10^–3^w/w) and homogeneously distributed (50–100 nm separation) in a ∼500 nm thick crystal of their monomer; the size of the domains within the macroscopic crystal are ∼20 μm^2^ (see Supporting Information (SI) Section S1). We refer to these samples henceforth as (thin) films. PDAs have found widespread use in a range of optoelectronic applications such as temperature sensors,^21^ lithographic resists,^22^ dye sensitized solar cells,^23^ photoelectric devices^24^ and transistors. Although PDA has found less use than other conjugated polymer systems such as P3HT or polyfluorene the absence of significant interchain disorder, cross-linking, and integration into nanostructures allows the theoretical properties of correlated 1D electronic systems to be tested in the *idealized* polymer limit in PDA, as such it is a model system to investigate exciton transport in polymer systems. The electronic structure of PDAs are very similar to other modern conjugated polymers such as polyacenes, iso-indigo-based polymers,^9,10,25^ carotenoids^26^ and singlet-fission materials making the results here applicable to many other systems. The torsional backbone angle is controlled by the side-chains of the diacetylene monomer and exact synthetic preparation method as shown in a previous study.^27^ Other than the backbone torsion both PDAs show near-identical structural and chemical material properties, that is, degrees of polymer content, polymer alignment, structural disorder, etc. (SI, Figure S1). Electronically, both PDA phases display strong coupling between the monomer units and exhibit large exciton coherence lengths.^20,27−29^ Owing to the planar backbone geometry, “blue” PDA exhibits an optically accessible 1^1^B_u_^+^(S_2_) which lies above a 2^1^A_g_^–^(S_1_) state. This ordering gives rise to a broad visible absorption spectrum dominated by vibrational progressions at 1400–1500 and 2100 cm^–1^ (C=C and C≡C stretching mode, blue spectrum; Figure 1b). By contrast, the energetic ordering in “red” PDA is reversed due to backbone torsions, resulting in a dark 2^1^A_g_^–^(S_2_) state lying above the optically accessible 1^1^B_u_^+^(S_1_) state. As a consequence, “red” PDA exhibits a significantly blue-shifted absorption spectrum compared to “blue” PDA (Figure 1b).

The ensemble excited state properties of “blue” and “red” PDA films following photoexcitation with a 10 fs pump pulse centered at 560 nm^30^ are shown Figure 1c,d. In “blue” PDA, the pump–probe spectrum is characterized by a sharp stimulated emission (SE) band at 650 nm, a broad photoinduced absorption (PIA) at 750–900 nm and a second narrower PIA at 680–700 nm (Figure 1c). By using spectral decomposition methods outlined in a previous study^31^ and fitting the electronic decays (exponential decay convoluted with instrument response) we find that the SE (1^1^B_u_^+^) decays monoexponentially to the ground state with a lifetime of 630 ± 10 fs, while the PIA at 750–900 nm decays with a lifetime 1300 ± 40 fs and the PIA at 680–700 nm with 2300 ± 130 fs. The observed excited-state features and dynamics agree excellently with previous reports.^20,31,32^ Following this agreement, we can assign the SE band to 1^1^B_u_^+^ (S_2_), the broad PIA is attributed to the 2^1^A_g_^–^(S_1_) state and the narrow PIA at 680–700 nm to a hot, vibrationally excited, ground state.^32−34^ Photoexcitation of “blue” PDA to 1^1^B_u_^+^ (S_2_) is thus followed by rapid internal conversion within 90 fs to 2^1^A_g_^–^(S_1_) (i.e., rise time for 2^1^A_g_^–^ kinetic as derived following spectral decompostion^31^) which decays back to the ground state. In “red” PDA the transient absorption spectrum shows a single broad PIA in the 650–900 nm range. The decay of this PIA is 13.8 ± 0.2 ps and has been suggested to correspond to the transition between 1^1^B_u_^+^(which is theS_1_ state in this system) and a state lying at ∼3–3.5 eV, of ^n^A_g_ or charge transfer (CT) character.^27^ We emphasize that we carried out both fs-TA (and fs-TAM measurements, see below) as a function of carrier density (excitations per unit length of polymer chain (*n*_0_) in a range from *n*_0_ = 0.1–2 nm^–1^; SI Section S4). In both cases these results showed that for “blue” PDA the kinetics do not vary with carrier concentration whereas annihilation was observed for “red” PDA.

In order to extract the exciton diffusion characteristics of “blue” and “red” PDA we performed widefield femtosecond transient absorption microscopy (fs-TAM) with a similar 10 fs pump pulse centered at 560 nm.^17,19,35^ In fs-TAM a pump pulse is focused via a high-numerical aperture (NA = 1.1) objective to near the diffraction limit Gaussian spot (σ = 143 nm), which locally creates excitons within the sample. After a variable time delay, a broadband, 7 fs probe pulse centered at 780 nm is loosely focused on the sample to image the pump-induced signal changes in the material. The transmitted probe is subsequently imaged onto a two-dimensional detector. By recording the transmission image with and without the pump pulse incident on the sample, we calculated the spatially resolved transient absorption image of the locally excited exciton distribution as a function of pump–probe time delay. Tracking the temporal evolution then allows us to extract information about exciton diffusion properties. For “blue” PDA we probed in the center of the 2^1^A_g_^–^ PIA band at 800 nm (blue stripes in Figure 1c), while the probe wavelength in “red” PDA was set to 790 nm to be centered with the 1^1^B_u_^+^ PIA (orange stripes in Figure 1d). Spectral decomposition based on previous studies^31^ demonstrates that this wavelength ensures that we exclusively monitor the S_1_ exciton population, free of other spectral features. This point is discussed further in SI Section S2 where we report the probe wavelength dependence of the fs-TAM demonstrating the “hot” exciton and S_1_ diffusion properties to be distinct. Although “blue” PDA undergoes faster electronic relaxation as compared to its “red” counterpart it is important to note that the electronic relaxation and diffusion coefficient are not necessarily linked, that is, dark channels, internal conversion, etc, will change the lifetime but not necessarily the diffusion coefficient.

**Figure 2:**
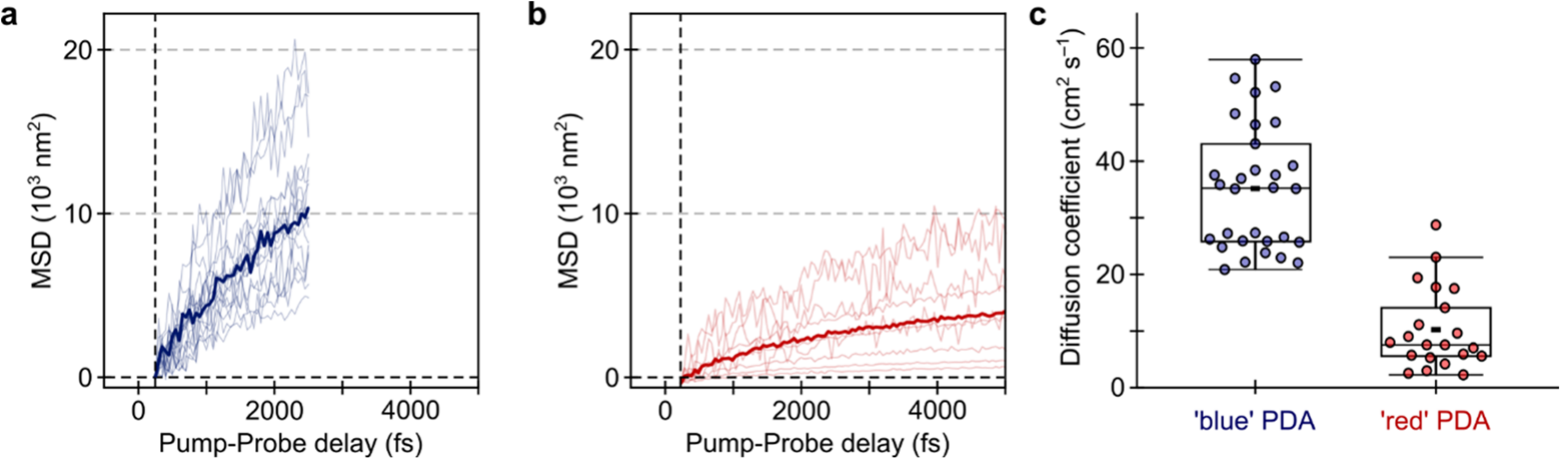
Femtosecond transient absorption microscopy results for “blue” and “red” PDA. a,b. MSD curves for “blue” (a) and “red” PDA (b), respectively. Solid line shows average curves whereas faint lines are from individual sample locations. Due to the faster decay in “blue” PDA, the MSD was only recorded to 2.3 ps. c. Scatter-box plot of diffusion coefficients for “blue” and “red” PDA. Boxed area is 25–75% range, with horizontal line representing median and whiskers for interquartile range. Filled black rectangles represent the mean.

To quantify the spatial extent of the signal, we extracted the spatial standard deviation for all transient absorption images using a 2D Gaussian fit (see SI Section S3). We subsequently computed the mean-square-displacement (MSD) for the underlying spatial exciton profile, which is a measure of the change in the spatial extent of the exciton population as a function of time. Fitting is performed with a 2D Gaussian as opposed to Bessel function (which results from the far-field radiation pattern of a transmitted plane-wave probe). This is due to the computational complexity associated with the latter and the fact that fitting with a 2D Gaussian shows that the diffraction rings are reproduced faithfully. This implies that the deviation from a true two-dimensional spatial profile in our measurements, due to the diffraction ring, has no effect on the retrieved spatial standard deviation (see SI Section S3). As shown in Figure 2a,b, both samples exhibit a pronounced increase in their MSD over the first 5 ps with “blue” PDA displaying MSD values exceeding “red” PDA. In both PDA phases, the exciton propagation is highly anisotropic and proceeds exclusively along a single axis (see SI Section S3). This behavior is expected based on the one-dimensional structure of PDA and the low polymer content (10^–4^ – 10^–3^w/w) of the samples remains, preventing interchain transport. Several chains will be excited by the pump pulse with the pump probe signal taken as the average of all chains within the exciting spot. Our results therefore suggest that exciton propagation proceeds along the long-axis of the PDA chains (monoclinic *b* axis of crystals).^20 ,27^ We remark that varying the probe wavelength within the broad PIA did not alter the observed transport behavior (SI Section Figure S2).

Although the PDA chains are highly ordered, their thin films are known to contain some underlying nanoscale inhomogeneity. We therefore repeated fs-TAM measurements on 30 sample locations in “blue” PDA and 21 in “red” PDA, to build up a statistical picture of singlet exciton transport. Furthermore, to accurately compare the spatial dynamics of both samples, we restricted our analysis to time-delays >250 fs in order to avoid effects due to S_2_–S_1_ internal conversion processes in “blue” PDA.

**Figure 3:**
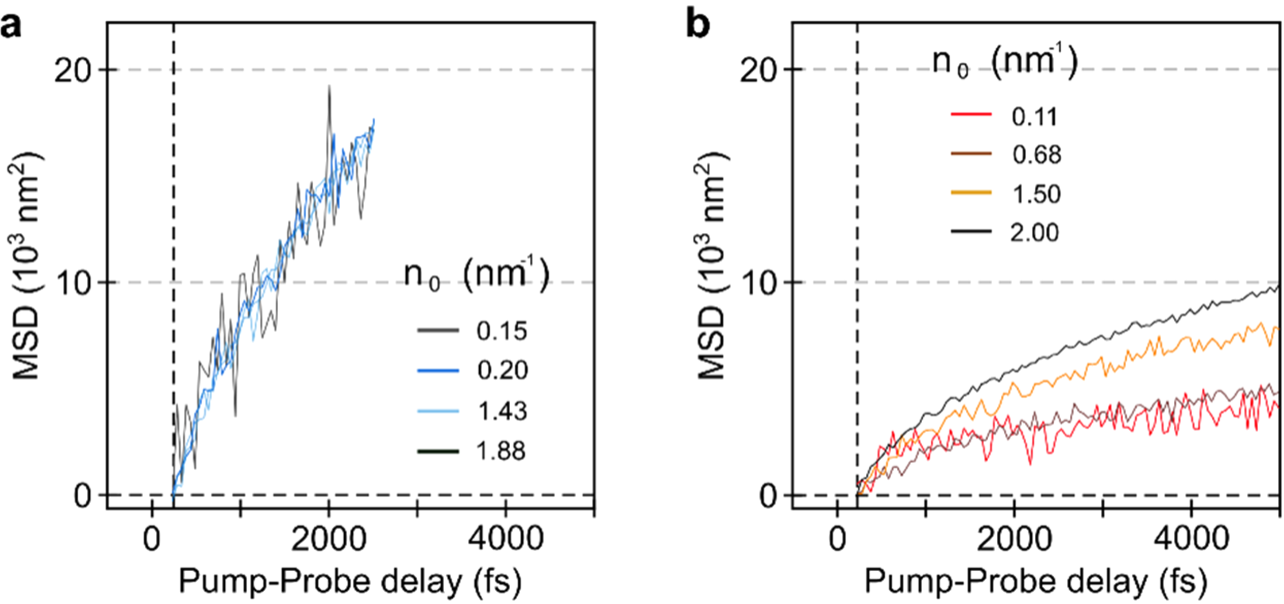
Femtosecond transient absorption microscopy as a function of carrier density for “blue” and “red” PDA. a,b. In the case of “blue” PDA (a) there is no dependence of the MSD on the carrier density, whereas in “red” PDA (b) the diffusion coefficient increases at higher pump fluences. In this latter case exciton–exciton annihilation effects can be considered to be playing a role. *n*_0_ represents the number of (initial) excitations per nm of polymer chain.

A salient feature of the MSD curves displayed in Figure 2a,b is that for “blue” PDA the maximum MSD value reached is typically greater than “red” PDA. In order to extract the relevant diffusion coefficients, we constructed the associated differential diffusion equation^36^
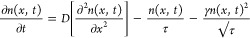
1Here *n*(*x, t*) is the electronic population at a time *t* and position *x*, *D* is the diffusion coefficient, τ is the electronic lifetime and γ is a coefficient which accounts for exciton–exciton annihilation. In the absence of exciton annihilation this equation can be solved to show that in one dimension,
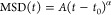
2where *A* is proportionality factor and α accounts for nonlinearity in time. In the case of α = 1 we retrieve normal diffusion, whereas α > 1 is known as superdiffusive behavior and α < 1 is referred to as subdiffusive behavior. In this latter case a time-dependent diffusivity *D*(*t*) can be defined as,
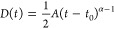
3In the presence of annihilation no analytical solution exists and eq 1 must be fit to extract *D* and γ^36^ (see SI Section S3 for further details). We emphasize that in the data shown in Figure 2,3, α is an empirical fix applied to the equation to best match the experimental data as opposed to being derived from the analytical solution to eq 1.

To identify, which model should be applied, we carried out a fluence dependence on the same sample spot for both PDAs, as shown in Figure 3. Here, we find that the transient spatiotemporal dynamics of “blue” PDA do not vary with power (Figure 3a), while “red” PDA displays a pronounced power dependence (Figure 3b), indicative of a higher annihilation rate. This increased annihilation rate of 1^1^B_u_^+^ compared to 2^1^A_g_^–^ excitons is an important observation that is not well understood. While beyond the scope of this article, we note that the 2^1^A_g_^–^ state exhibits a lower transition dipole moment than 1^1^B_u_^+ 7^,.^37^ Given that the annihilation cross-section of a state is proportional to its transition dipole moment^38−40^ this may explain why the 2^1^A_g_^–^ symmetry excitons do not exhibit significant annihilation effects.^41^ A similar effect has been observed in H- and J-aggregate systems. In the former, where S_1_ is dark, the phase relationship between two dipoles leads to destructive interference and suppressed annihilation, whereas in J-aggregates where S_1_ is bright the opposite occurs.^41−43^ The 2^1^A_g_^–^ excitons can still scatter in such a way that would lead to annihilation (impact ionization, Auger effects, etc), but this would require the excitons to be very close, or at high densities. We note that it is possible that local geometry/structure around the excitons prevents them from approaching each other, and that the excitation density does not reach the magnitudes required for short-range annihilation mechanisms to be operative (on the time scales of internal conversion). Computing the interexciton separation (at the highest excitation densities used in the study) shows that excitons remain separated by at least 1 monomer unit in both “blue” and “red” PDA suggesting Auger and impact ionization events can indeed be ignored (see SI Section S5). Other effects such as disorder or local microstructure may in addition to the symmetry of S_1_ play a role in suppressing annihilation in “blue” PDA. Study of other polyene systems such as carotenoids which have been shown to have similar dynamics to PDA,^31^ may aid this, however independently investigating the effects of symmetry remains challenging.

Based on the observed power dependence, we applied eq 1 to directly extract diffusion constant, *D*, and annihilation parameter, γ for “red” PDA. In contrast, for “blue” PDA we fit eq 2 to the MSD trace to extract *A* and α and then extract the diffusion coefficient from the time-dependent diffusivity *D*(*t*) according to eq 3. Following this analysis, we derive a mean diffusion coefficient of 34 ± 10 cm^2^ s^–1^ for “blue” PDA (value at 2500 fs, see SI Section S3 for full *D*(*t*)), which is significantly larger and more varying across different sample locations as compared to “red” PDA with 7 ± 6 cm^2^ s^–1^. We remark that the spread in diffusion coefficients for “blue” PDA is larger than for “red”, however further studies are required to ascertain the origin of such behavior. The exponent value, α, for “blue” PDA lies between 0.7–0.9 and indicates subdiffusive exciton transport. For “red” PDA the annihilation coefficient, γ, ranged between 0.1–1.1 cm s^–0.5^ (SI Section S5). The units of cm s^–0.5^ reflect the one-dimensional nature of polymer. The annihilation coefficient, like the diffusion coefficient, will be time dependent, however is found not to vary in the time range studied here nor is found to vary significantly with sample location (SI Section S5). We note that to obtain *D* for “red” PDA the fluence dependent traces were fit independently. The data in Figure 2 captures the heterogeneity (between sample locations) at a single fluence. The variation in the annihilation coefficient is further captured by repeating fluence dependent measurements at four different sample locations; γ is found to have a maximum spread of 0.21 cm s^–0.5^.

Critically, while this annihilation factor is substantial, the ratio of the first and third term in eq 1 is ∼3–5 at early times, rising to ∼10–20 after 3 ps (SI Section S5). This suggests that the annihilation is still only a minor contributor to the subdiffusive transport.^44^ In comparison to other conjugated polymer systems the annihilation coefficient in “red” PDA is around 10 times lower, for example, as compared to polyfluorene^45^ where γ was measured to be ∼3 cm s^–0.5^. However, γ is also 2–3 orders of magnitude larger than in these systems^1^ and more comparable to that observed in 2D TMDC materials such as MoS_2_,^46^ black phosphorus^47^ and 2D perovskites.^48^ Comparison of absolute values should, however, be performed with care due to the time dependent nature of the diffusion coefficient and the difference in dimensionality between these systems. At early times (sub-1 ps) γ will be higher due to the radiative decay of excitons, the *t*^–1/2^ dependence of the annihilation parameter and static annihilation; we do not consider this latter contribution at the fluences used here in-line with other studies.^45^ We note for “red” PDA, the diffusion coefficients extracted in Figure 2c are from traces at the lowest fluence used in this study (*n*_*0*_ (carriers per unit length of polymer) ∼ 0.1 nm^–1^).

## Discussion

Having discussed the origin of the observed exciton dynamics we can determine the overall diffusion length from
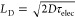
5where *τ*_elec_ is the intrinsic lifetime of the underlying process. Previously we have determined for 2^1^A_g_^–^, τ_elec_ is ∼600 fs, whereas for 1^1^B_u_^+^,τ ∼ 9000 fs.^31^ Based on these values we can extract diffusion lengths of 26 ± 6 nm and 33 ± 5 nm, for “blue” and “red” PDA, respectively. It is noteworthy to mention that the diffusion length is similar for both systems, despite their stark difference in diffusion coefficient and annihilation characteristics. Long-range exciton transport thus requires not only highly mobile excitons with minimized annihilation, but also long state lifetimes. We re-emphasize at the polymer concentrations used here, the PDA chains do not interact, with the separation between chains (∼100 nm) being larger than the exciton diffusion length. Indeed, several studies have shown that interchain hopping and the formation of interchain charge transfer states does not occur in PDA.^49,50^ In a previous study it was also shown that exciting at the band-edge of PDA, whose band-edge absorption is dominated by partially polymerized states, does not show any new spectroscopic signatures or electronic lifetime changes.^31^ This again furthers the conclusion that hopping between chains does not occur. Finally, the low temperature absorption line width of crystals studied here is similar to that reported for isolated chains in previous studies (SI Section S1), this again suggests that interchain interactions are not significant.^51^ These observations further corroborate the claim that defect states generally play little role in our observations here. Performing fs-TAM measurements on thicker PDA crystals which have a lower Urbach energy^31^ (30 meV versus 34 meV) shows that the diffusion coefficient and dynamics remain qualitatively unchanged (SI Section S4). There is however a small difference (0.6–2 cm^2^ s^–1^) in the diffusion coefficient between the thick and thin crystals which may be related to the ∼10% difference in electronic disorder; further studies are needed to fully confirm this. We finally also note that PDA crystals were masked under a polarized optical microscope prior to measurement (SI Section S1) to ensure excitation was performed away from grain boundaries which can be well resolved in the materials. As such although the energetic disorder is larger than *k*_B_*T* in the materials our results suggests that trap states are not significant in our observations or responsible for the difference in transport between “blue” and “red” PDA.

Despite the high diffusion coefficients obtained at room temperature in these systems (Figure 2), which would be suggestive of a strong tendency of excitons to delocalize, we observe subdiffusive exciton transport in both 2^1^A_g_^–^ and 1^1^B_u_^+^ states, which is typical of strong exciton–phonon interactions^52^ that lead to localized excitons. In order to understand these seemingly inconsistent observations, we employ ab initio *GW*-BSE calculations to quantify the two competing effects. These calculations on the properties of excitons and exciton–phonon interactions have previously been systematically tested on PDAs as a part of a separate study.^53^ The electronic coupling between excitons residing on neighboring monomers of the polymer chain (*J*-coupling) drives the system to a more delocalized state. From a calculation of the exciton bandwidth (the range of the energy-momentum dispersion along the chain, SI Section S6) we could determine *J* = 0.54 eV for the 1^1^B_u_^+^ state of the planar (“blue”) PDA. Unfortunately, the multiexcitonic character of the 2^1^A_g_^–^ state prevents us from obtaining the corresponding *J* value within *GW*-BSE, which is a methodology based on Green’s functions that only captures single excitations^54^ and cannot describe biexcitons.^55^ Extending this formalism to describe excited states involving more than two particles is an active field of research and beyond the scope of this work. Nevertheless, the larger diffusion coefficient of the 2^1^A_g_^–^ state makes it likely that it will have a value of *J* that is greater than 0.54 eV. The 1^1^B_u_^+^ state of the “red” PDA will have a smaller excitonic coupling compared to the same state in the “blue” phase due to the decreased p-orbital overlap that the torsion θ between subsequent monomers induces, and in particular a simple estimate of its value is *J*·cos θ = 0.41 eV. We note that while the 2^1^A_g_^–^ state has been shown to possess ^1^(TT) character, no free triplets^31^ have been observed to form in the specific type of red or blue PDA crystals studied here, hence we do not consider their contribution here. This in accordance with the low triplet yield via singlet fission in PDA^33,56^ and the generally low intersystem crossing yields in conjugated polymers.^57^ This in agreement with the significantly lower diffusion coefficient reported for triplet excitons^58,59^ (2–3 orders of magnitude lower than observed here for singlets) and predictions of a lower J coupling.^55^

Moreover, we quantify the strength of exciton–phonon coupling by calculating the reorganization energy of the 1^1^B_u_^+^ state of PDA to be λ = 0.43 eV (SI Section S7), which denotes the driving force of the system to localize after photoexcitation. The carbon–carbon double- and triple-bond stretch have the largest contribution to the reorganization energy (see Huang–Rhys factor, SI Table S2), resulting in the prominent vibronic progression of the absorption spectrum of Figure 1b. This can be intuitively understood from the fact that these vibrational motions allow the polymer chains to transiently explore configurations closer to the limit of a structure without Peierls distortion and hence to the metallic limit.^60^ Indeed, the exciton wave function is significantly affected when these vibrations are displaced compared to other motions, as shown in SI Figure S6. Moreover, phonons are known to lead to a renormalization of exciton properties compared to a “static” picture.^61^ In order to quantify the vibrationally induced renormalization of properties relevant to exciton diffusion in PDA we employ a quadratic approximation (SI Section S7), which allows us to identify the effect of the individual phonon modes. We find that phonons at room temperature only lead to a small increase of the exciton energy by 15 meV, and a small decrease of the excitonic coupling by 5 meV. However, we find that the magnitude of the exciton transition dipole moment undergoes a more significant renormalization due to the effect of phonons, from a value of 1.82 au to 1.30 au, and it is mainly the carbon–carbon double- and triple-bond stretching motions that drive this effect (SI Table S3). This reduction in the magnitude of the transition dipole moment |**μ**| results in reduced exciton–exciton annihilation, since the cross-section of this process is proportional to |**μ**| as already discussed previously. Hence phonons appear to quench the competing to exciton diffusion effect of exciton–exciton annihilation.

Based on the reorganization energy and *J*-coupling strength, we can now classify the transport regime in which PDAs operate. If *J*-coupling is small compared to the reorganization energy, we anticipate the system to localize on a single monomer and transport will only occur via incoherent hopping. Conversely, in our PDA films, we calculated, *J* ∼ λ. This suggests that exciton transport may operate in the coherent regime where exciton delocalization is dominant.^62,63^ However, the similarity between *J* and λ also suggests that exciton localization via exciton–phonon couplings can still occur. The subtle interplay between exciton–phonon coupling (localization) and *J*-coupling (delocalization) demonstrates that exciton transport occurs through the motion of partially delocalized excitons that reside on several monomers. This leads to subdiffusive exciton transport with high diffusion coefficients. We expect this conclusion to carry to the 2^1^A_g_^–^ state, since the *J* coupling of this state is likely larger than that of 1^1^B_u_^+^, as its experimentally measured more rapid delocalization suggests. Moreover, increasing exciton delocalization has been shown to lead to reduced coupling to high-frequency phonons in organic materials.^61^ Since the reorganization energy is dominated by the contribution of such high-frequency modes (SI Table S2), we expect its value to be smaller for the 2^1^A_g_^–^ compared to the 1^1^B_u_^+^ state, and for the criterion *J* > λ that defines coherent transport to still hold.

## Conclusion

In summary we have investigated the influence of electronic state symmetry on exciton diffusion in conjugated polymers. We have shown 2^1^A_g_^–^ excitons have on average ∼3 times higher diffusion coefficients than 1^1^B_u_^+^ excitons in PDA polymer chains. Where the exciton transport of 1^1^B_u_^+^ excitons exhibits annihilation effects, for 2^1^A_g_^–^ excitons, the smaller transition dipole moment likely results in no measurable annihilation. The motion of both 2^1^A_g_^–^ and 1^1^B_u_^+^ excitons however appears to remain subdiffusive likely due to exciton–phonon mediated trapping effects. Despite its smaller diffusion coefficient, the longer electronic lifetime of 1^1^B_u_^+^ means that 2^1^A_g_^–^ and 1^1^B_u_^+^ excitons have a similar diffusion length (∼30 nm). Our results suggest that 2^1^A_g_^–^ excitons are able to move across space as effectively as excitations that live orders of magnitude longer. This is in part due to the absence of annihilation losses.

The polymer backbone geometry will also play a role, that is, “blue” PDA is planar, whereas the “red” PDA is twisted. This will also influence coupling across monomers along the chain via orbital overlap and the extent of exciton delocalization. However, these effects must be combined with electronic correlation phenomena, such as the formation of the 2^1^A_g_^–^, such that a simple band picture with different hopping matrix elements cannot explain all of our observations. Indeed, it is not obvious that the dispersion (which will determine the group velocity and later the diffusion rates) is necessarily larger in the “blue” PDA, compared to the “red”. For example, if 2^1^A_g_^–^ has a strong triplet-pair contribution, as some studies have suggested, one might even imagine that the excitation would spread more slowly, even though the monomers have stronger electronic coupling.

In terms of design rules for organic electronics our results highlight a number of key principles. First, even in highly ordered systems introducing a small amounts of electronic disorder can potentially affect the transport behavior as evidenced by measurements on PDA crystals of different thickness. Additionally, the results suggest that not only delocalization (via the backbone twist angle) is important in enhancing exciton diffusion but the symmetry of S_1_ should be considered when designing new conjugated polymers for exciton transport both in terms of annihilation and diffusion properties. Our results describing the interplay of electron–phonon coupling and *J*-coupling suggest that careful consideration of bonding motifs should be taken such that the contribution of both to transport can be optimized. Finally, although PDA has not found as widespread used in organic electronics as other semiconducting polymers our results with high diffusion coefficients call for a renewed interest in the material.

## Methods

### Sample Preparation

#### “Blue” Polydiacetylene

3BCMU (3-methyl-*n*-butoxy-carbonylmethyl-urethane) diacetylene molecules were synthesized in-house using the method previously outlined by Se et al. and references therein.^64^ The synthesis classically consisted of two steps: (i) oxidative coupling of 4-pentyn-1-ol (Hay’s method) to produce the 4,6-decadiyn-1,10-diol and then, (ii) reaction of the diol with *n*-butylisocyanate acetate. Note that the source 4-pentyn-1-ol was not synthesized at the laboratory but purchased from Sigma-Aldrich (Merck). Ultrathin single crystals were grown between two coverslips using a melt-processing method. The whole process was systematically carried out under a polarized optical microscope so as to be able to follow and control the sample elaboration: a very small amount of diacetylene powder is placed at one edge of the double-slides assembly; when heating above the melting temperature (∼65 °C) the liquid diacetylene fills the empty space by capillary action to form a thin liquid film between the two substrates. Rapid cooling leads to the formation of a highly polycrystalline film. The sample is then heated again to around the melting temperature until the melting of all the crystallites took place. When only a few crystal germs remain the sample is cooled again at a very slow cooling rate (typically <0.1 °C/mn) to induce the growth of large single monocrystalline domains from the germs. Typical polymer contents by weight are then in the 10^–4^–10^–3^ range. The interchain separation (∼100 nm; homogeneous distribution) is obtained from the absorption optical density (OD) and Beer Law, where  where ∝is taken as ∼1 × 10^6^ cm^–1 20^ for light polarized parallel to the long axis of the chains and *l* (the thickness of the crystal) is ∼500 nm. The spatial separation between chains is assumed to be significantly large enough that there are negligible interchain interactions. X-ray diffraction^31^ and microscopic optical transmission measurements (SI Section S1) also confirm that chains remain linear and highly aligned over macroscopic length scales, again suggesting that interchain interactions can be ignored.

#### “Red” Polydiacetylene

To make multipulses microscopy possible by limiting scattering of the pump pulse ultrathin crystalline monodomains of the (“red” PDA: 3Nφ_2_) diacetylene were grown before being slightly polymerized. Large size crystalline regions (∼mm^2^) with submicronic thicknesses (∼100–200 nm) are obtained using a melt processing method.^65^ A few mg of the purified diacetylene powder are melt (*T* ≥ 80 °C) and the liquid is injected, using capillarity action, in the space between two superimposed microscope coverslips. After rapid cooling the thin liquid film crystallization leads to the formation of a highly polycrystalline structure, being the assembly of microdomains. The sample is then heated again under a polarized optical microscope until almost all the melting takes place but preserving a few single crystal germs. At that point the sample is cooled again at a very slow cooling rate to induce the growth of hundreds of μm–mm scale domains from the germs until room temperature is reached. The (3Nφ_2_) is a highly stable monomer that is characterized by both weak thermal- and weak UV photo- reactivity.^27^ Polymer chains are thus generated by exposure to X-ray light in a diffractometer (Rigaku, Smartlab). The polymer content is adjusted by trial and error and kept low enough (typically below 0.1% in weight) to form a solid solution of isolated polydiacetylene chains inside their diacetylene host crystal.

### Absorption Spectroscopy

Polarized absorption spectroscopy of PDA crystals was performed using a home-built setup with a white light source generated by focusing the fundamental of a Yb-based amplified system (PHAROS, Light Conversion) into a 4 mm YAG crystal. The resulting absorption (corrected for the sample substrate) was then collected by imaging with a Silicon photodiode array camera (Entwicklunsbüro Stresing; visible monochromator 550 nm blazed grating).

### Femtosecond Pump–Probe Spectroscopy

The fs-TA experiments were performed using a Yb-based amplified system (PHAROS, Light Conversion) providing 14.5 W at 1030 nm and 38 kHz repetition rate. The probe beam was generated by focusing a portion of the fundamental in a 4 mm YAG substrate and spanned from 520 to 1400 nm. The pump pulses were generated in home-built noncollinear optical parametric amplifiers (NOPAs). The NOPAs output (∼4 to 5 mW) was centered typically between 520 and 560 nm (fwhm ∼65–80 nm), and pulses were compressed using a chirped mirror and wedge prism (Layerterc) combination to a temporal duration of ∼9 fs. Compression was determined by second-harmonic generation frequency-resolved optical gating (SHG-FROG; upper limit) and further confirmed by reference measurements on acetonitrile where the 2200 cm^–1^ mode could be resolved. The probe white light was delayed using a computer-controlled piezoelectric translation stage (Physik Instrumente), and a sequence of probe pulses with and without pump was generated using a chopper wheel (Thorlabs) on the pump beam. The pump irradiance was set to a maximum of 38 μJ/cm^2^. After the sample, the probe pulse was split with a 950 nm dichroic mirror (Thorlabs). The visible part (520–950 nm) was then imaged with a Silicon photodiode array camera (Entwicklunsbüro Stresing; visible monochromator 550 nm blazed grating). The near-infrared part was imaged using an InGaAs photodiode array camera (Sensors Unlimited; 1200 nm blazed grating). Measurements were carried out with a time step size of 4 fs out to 2 ps to minimize the exposure time of the sample to the beam. Unless otherwise stated, all measurements were carried out with the probe polarization set parallel with respect to that of the pump (using a half-waveplate; Eksma) and along the PDA chains. The absorption spectrum of samples was measured after each pump–probe sweep to account for any sample degradation.

### Femtosecond Pump–Probe Microscopy

The femtosecond wide-field detected transient absorption microscope has been described in detail previously.^19,35^ Briefly, a Yb:KGW amplifier system (LightConversion, Pharos, 5 W, 180 fs, 1030 nm, 200 kHz) was used to seed two white-light stages for pump and probe generation. The pump white-light (3 mm Sapphire) was spectrally adjusted with a 650 nm short-pass filter (FESH650, Thorlabs), and compressed to 10 fs for all optical elements with two pairs of third-order compensated chirped mirrors and a wedge-prism pair (Layertec). Subsequently, the mode of the pump pulse is cleaned by a pinhole before being focused through the objective lens (NA = 1.1, oil immersion) to a spot size of ∼340 nm (full-width-half-maximum). The probe white-light (3 mm YAG) was spectrally adjusted to 650–900 nm in a home-build fused silica prism filter and compressed to 7 fs with a pair of third-order compensated chirped mirrors and a wedge-prism pair (Venteon) before being free-space focused onto the sample (20 μm Gaussian spot size full-width-half-maximum). The transmitted probe was imaged onto an emCCD (Rolera Thunder, Photometrics) at 55.5 nm/pixel as verified by a resolution target. The frame rate of the camera was set to 30 Hz with an integration time of 11 ms and pump off/on images were generated by a mechanical chopper at a frequency of 15 Hz. For the measurements, we adjusted the pump fluence to achieve initial concentration of carriers per unit length of polymer (*n*_0_) in the range: *n*_0_ = 0.1–2 nm^–1^ for all samples.

## Supporting Information Available

Optical microscopy of PDA, photothermal deflection spectroscopy, TAM modeling methods, pump fluence dependence, annihilation in “blue” and “red” PDA, theoretical modeling (PDF)

## Notes

The raw data underlying this manuscript is freely available at https://doi.org/10.17863/CAM.66251.
