# Supplementary material for: Exciton Diffusion in Highly-Ordered One Dimensional Conjugated Polymers: Effects of Back-Bone Torsion, Electronic Symmetry, Phonons and Annihilation
Source: J Phys Chem Lett. 2021 Apr 8;12(14):3669–78. doi: 10.1021/acs.jpclett.1c00193 (PMC8154834; doi:10.1021/acs.jpclett.1c00193)
Supplement: Supplementary file 1 — jz1c00193_si_001.pdf [file jz1c00193_si_001.pdf]

Exciton Diffusion in Highly-Ordered One Dimensional Conjugated Polymers: Effects of Back-Bone Torsion, Electronic Symmetry, Phonons and Annihilation

[1]Raj Pandya*, [1]Antonios M. Alvertis, [1]Qifei Gu, [1]Jooyoung Sung, [2]Laurent Legrand, [3]David Kréher, [2]Thierry Barisien, [2]Alex W. Chin, [1]Christoph Schnedermann, and [1]Akshay Rao*

[1]Cavendish Laboratory, University of Cambridge, J.J. Thomson Avenue, CB3 0HE, Cambridge, United Kingdom

[2]Sorbonne Université, CNRS, Institut des NanoSciences de Paris, INSP, 4 place Jussieu, F-75005 Paris, France

[3]Sorbonne Université, CNRS, Institut Parisien de Chimie Moléculaire (IPCM) UMR 8232, Chimie des Polymères, 4 Place Jussieu, 75005 Paris, France

correspondance : *rp558@cam.ac.uk, ar525@cam.ac.uk

Supporting Information

Table of Contents

S1: Optical micrographs and photothermal deflection spectra of 'blue' and 'red' PDA 2
S2: Probe wavelength dependence of MSD in 'blue' and 'red' PDA 7
S3: Extraction of diffusion coefficients from fs-TAM data for 'blue' and 'red' PDA............................9
S4: Pump fluence dependence of fs-TAM dynamics of 'blue' and 'red' PDA....................................13
S5: Annihilation in 'blue' and 'red' PDA..15
S6: Calculation of the exciton properties of PDA 19
S7: Theoretical estimation of exciton-phonon effects in PDA 21
References...25

S1: Optical micrographs and photothermal deflection spectra of ‘blue’ and ‘red’ PDA

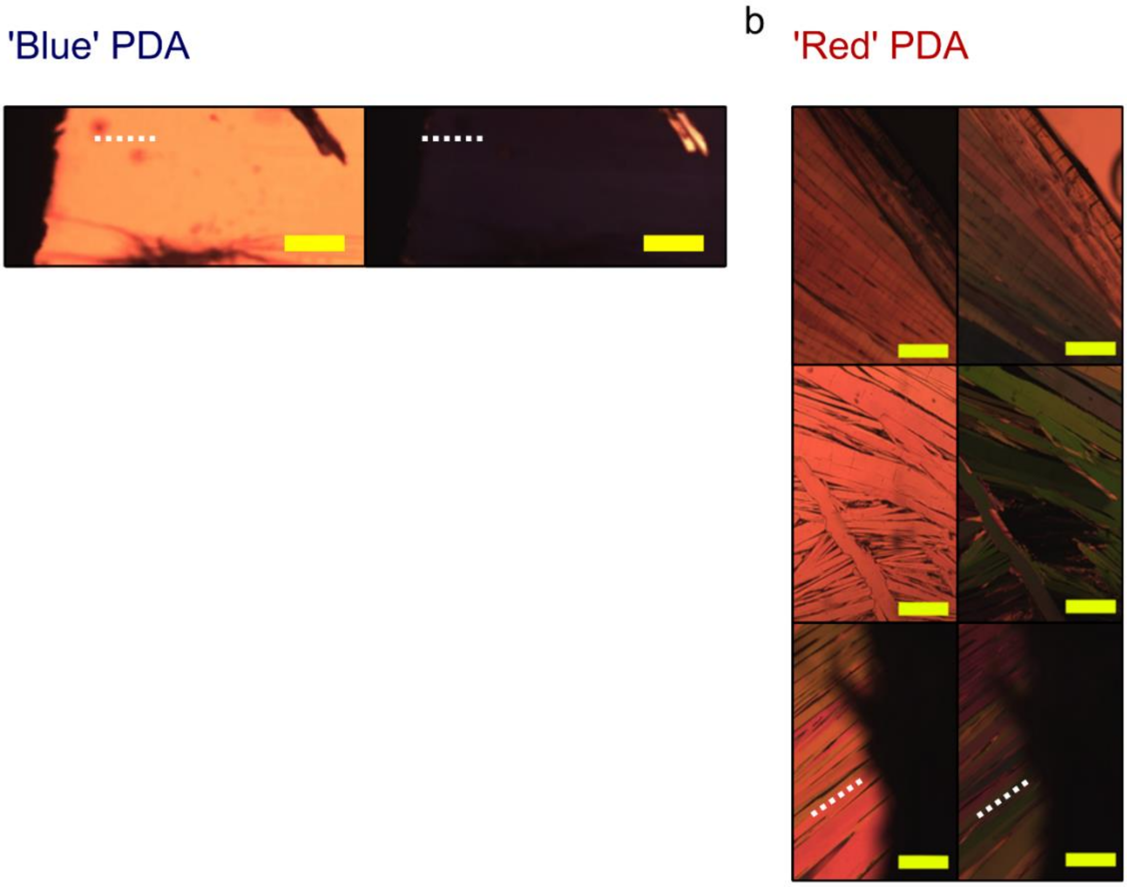

Figure S1: a-b. Optical micrographs of ‘blue’ and ‘red’ PDA. When the incident light is polarised perpendicular to the long-axis of the chains, there is full transmission (left side image). When the light is polarised parallel to the chain long-axis incident light is extinguished (right side image). Regions of the samples with high-degrees of polymer alignment are identified and masked before performing fs-TAM measurements. White dashes indicate line along which intensity is measured as a function of analyser angle in **Figure S1i**. Yellow scale bar is 2 μm.

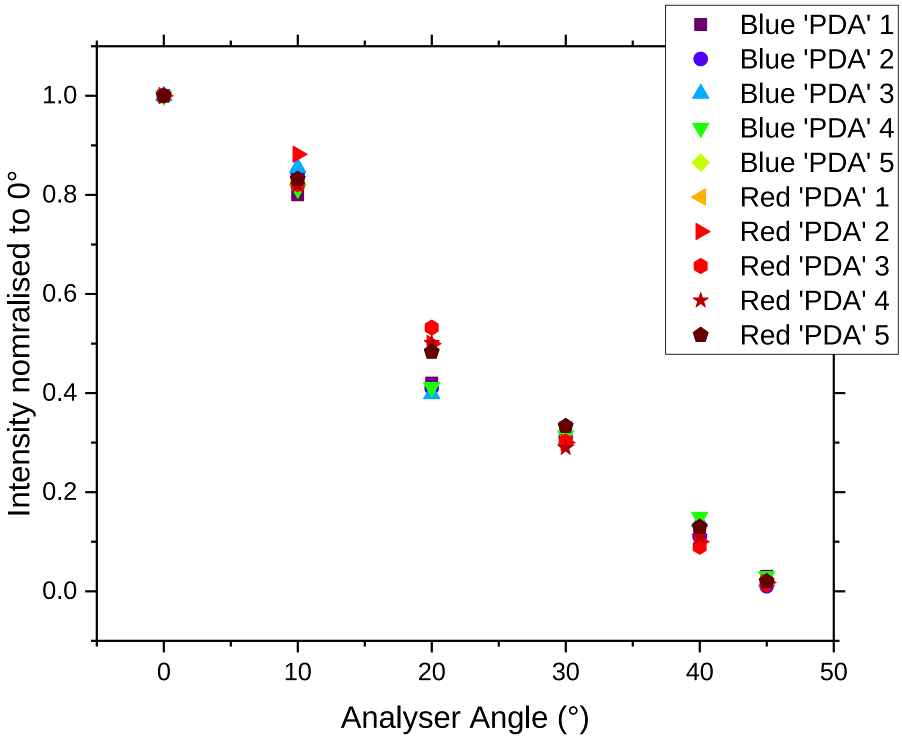

Figure S1i: Intensity of light along 2 um segment (5 sample locations; see **Figure S1i**) as analyser is rotated. Uniform extinction of the light is observed suggesting little bending and linear alignment of the chains.

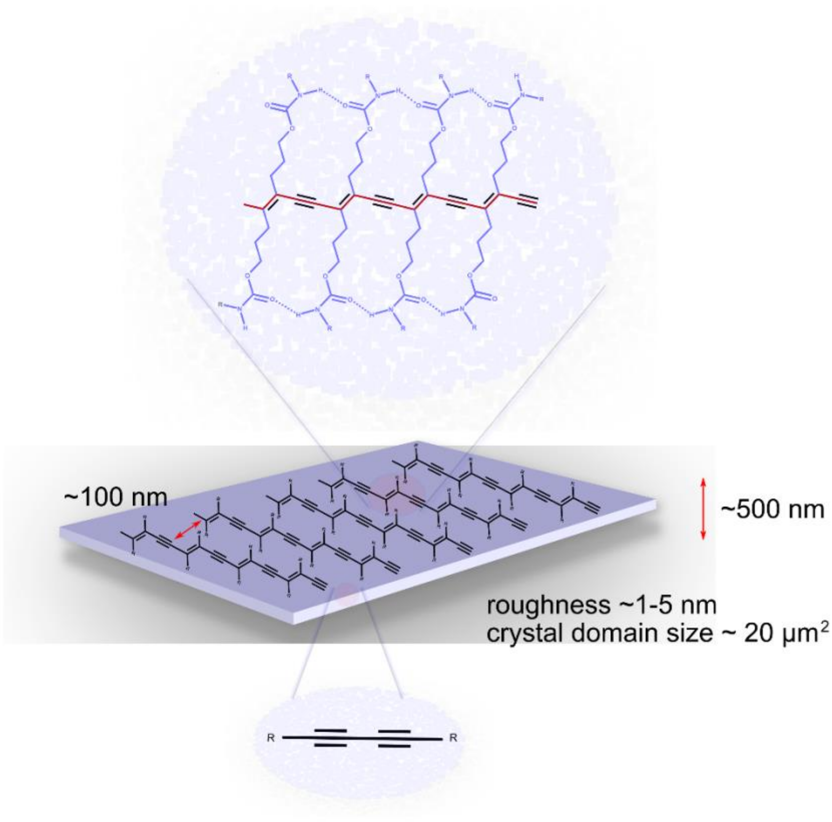

Figure S1ii: Cartoon highlighting the nature of the samples. PDA chains are homogeneously distributed in dilute concentrations in a crystal of their diacetylene monomer. The chains are separated by ~100 nm and the crystal is ~500 nm thick. The pump probe signal is taken as the average of all chains within the exciting pump-probe spot, with multiple PDA chains excited.

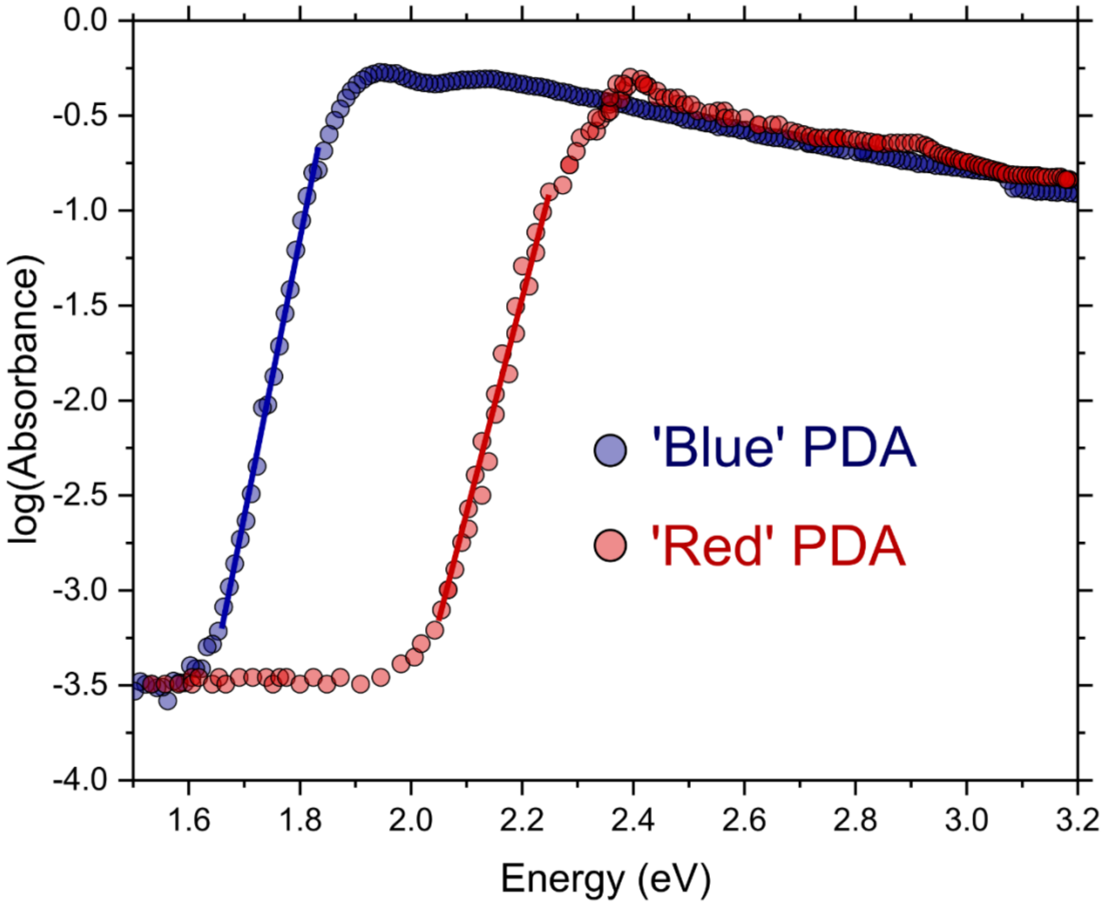

Figure S1iii: Photothermal deflection spectra of 'blue' and 'red' PDA. From the band-edge we can characterise the energetic disorder *via* the Urbach energy, E_u, which is related to the absorbance of the material, A, by $A(E) \propto e^{E/E_u}$, where E is the photon energy (solid line). Both samples have a similar of Urbach energy ~31 meV.

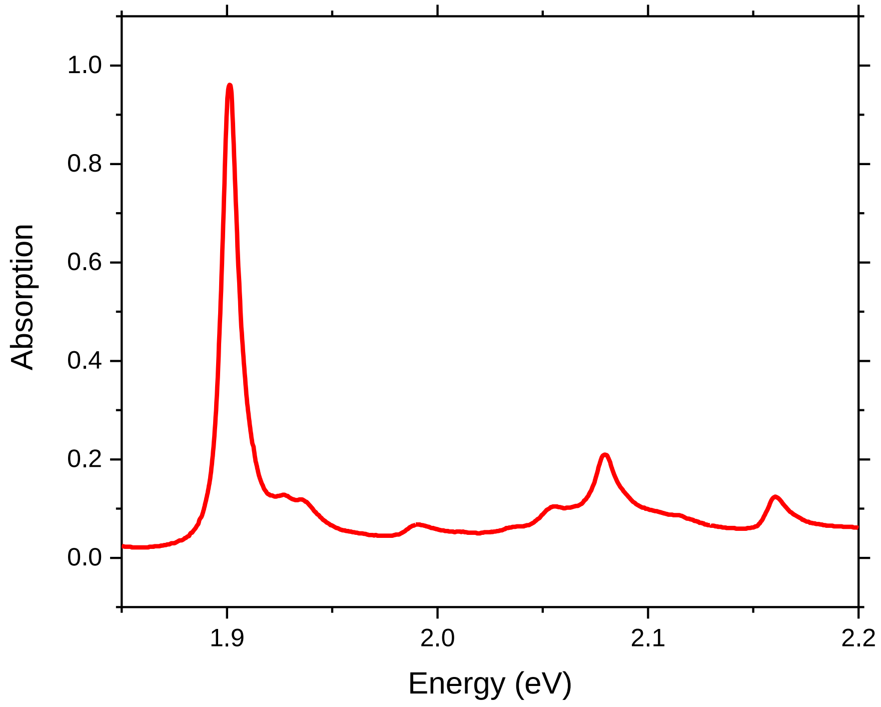

Figure S1iv: Absorption spectrum of 'blue' PDA at 4 K. The zero-phonon line at 1.9 eV has a linewidth of ~10 meV similar to that observed in previous studies for isolated chains[1].

S2: Probe wavelength dependence of MSD in 'blue' and 'red' PDA

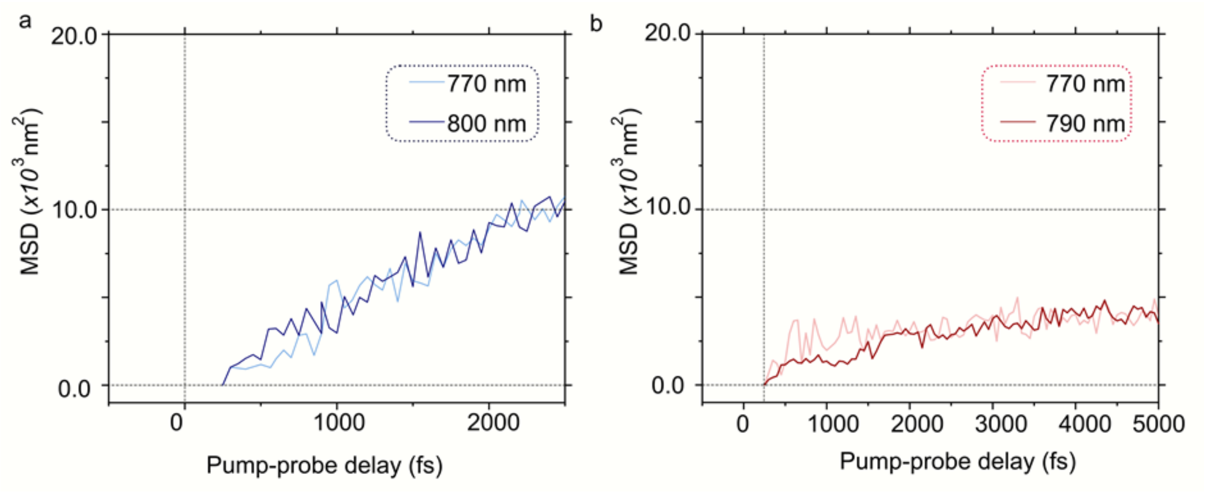

Figure S2: a-b. Probe wavelength dependence of fs-TAM dynamics for 'blue' and 'red' PDA. Measurements are taken at the same sample location and pump fluence with only the probe wavelength adjusted. As can be seen from the traces moving to bluer (shorter) probe wavelengths within the broad $2^1A_g^-$ and $1^1B_u^+$ PIAs does not alter the MSD. Due to experimental limitations wavelengths longer than 800 nm could not be studied.

To ensure there is no contribution from the 'hot' GS population to the diffusion of S_1 excitons fs-TAM measurements were repeated at 710 nm and 860 nm (same sample location). As shown in Figure S2i the 710 nm MSD trace shows markedly different evolution to that at 860 nm and 800 nm. In a previous study[2] the signal at 710 nm was shown to be dominated by a 'hot' GS population of excitons. The spectral overlap between the PIA associated with this state and S_1 is almost negligible (~12 %) at the probe wavelength of 800 nm used in our experiments. Hence we do not believe this state contributes significantly to the rapid expansion observed. Understanding the dynamics of the 'hot' ground state is beyond the scope of this work and reserved for future studies. We also note that in the previous works it was shown that 'hot' ground state evolves *from* S_1 after ~2 ps hence is unlikely to influence the early time behaviour we study here.

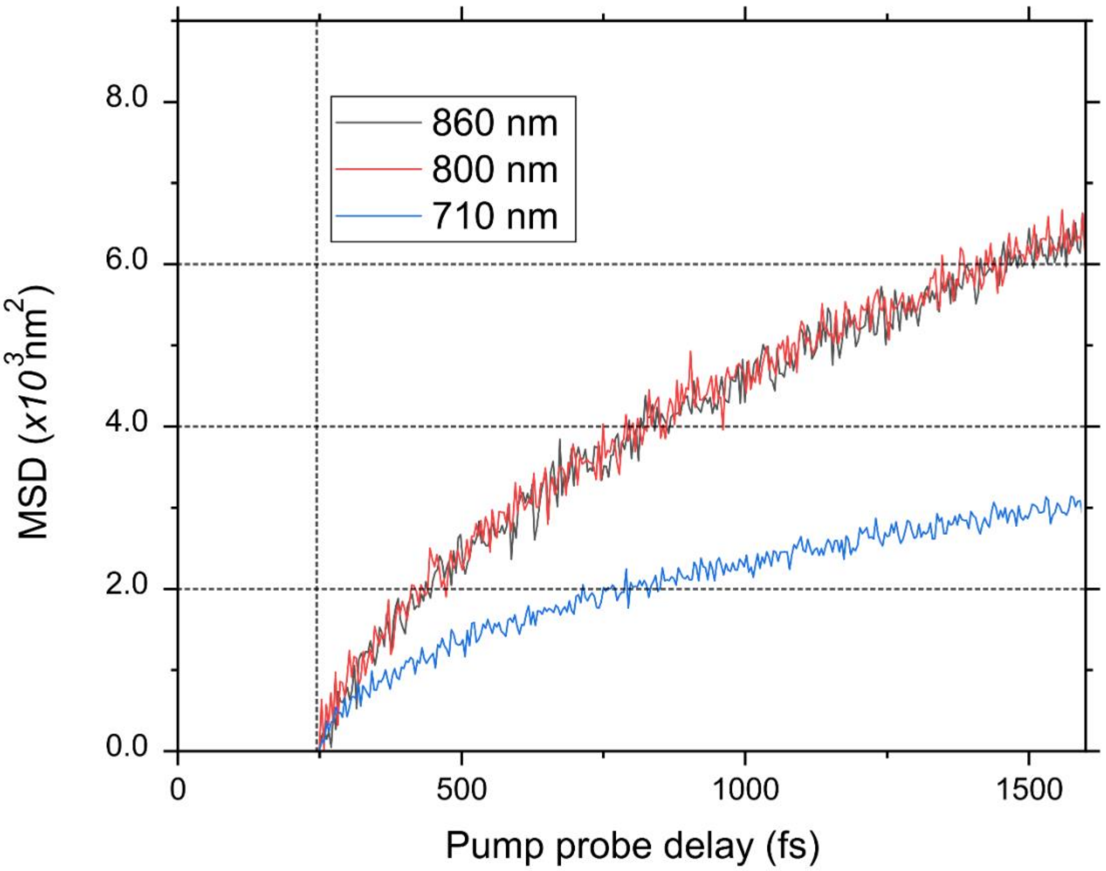

Figure S2i: MSD trace from fs-TAM experiments at 800 nm, 860 nm and 710 nm.

S3: Extraction of diffusion coefficients from fs-TAM data for 'blue' and 'red' PDA

For 'blue' PDA the diffusion coefficient was extracted as follows:

(i) fs-TAM images were fit with a 2D Gaussian function.
(ii) The Gaussian standard deviation (σ) was extracted for each image along the wire long- and short (principle) axes.
(iii) A mean-square-displacement was calculated for each image using MSD = $\sigma(t)^2 - \sigma(t_0)^2$.
(iv) The MSD was fit using equation 2 of the main text.
(v) A differential diffusion equation was calculated at the diffusion coefficient extracted from the value at 2500 fs.

For 'red' PDA exciton-exciton annihilation effects are present and the above method cannot be used to determine the diffusion coefficient. Instead the fs-TAM data was fit to equation 1 of the main text as follows:

(i) Derive initial parameters.
(ii) Numerically solve equation 1 of main text to data.
(iii) Fit Gaussian to solution at each time point.
(iv) Compute sum of squares of residuals between solution's Gaussian-fitted variance and data's Gaussian-fitted variance at each time point until error is sufficiently low.
(v) Extract MSD and annihilation coefficients.

All fitting was performed using the Mathematica software package.

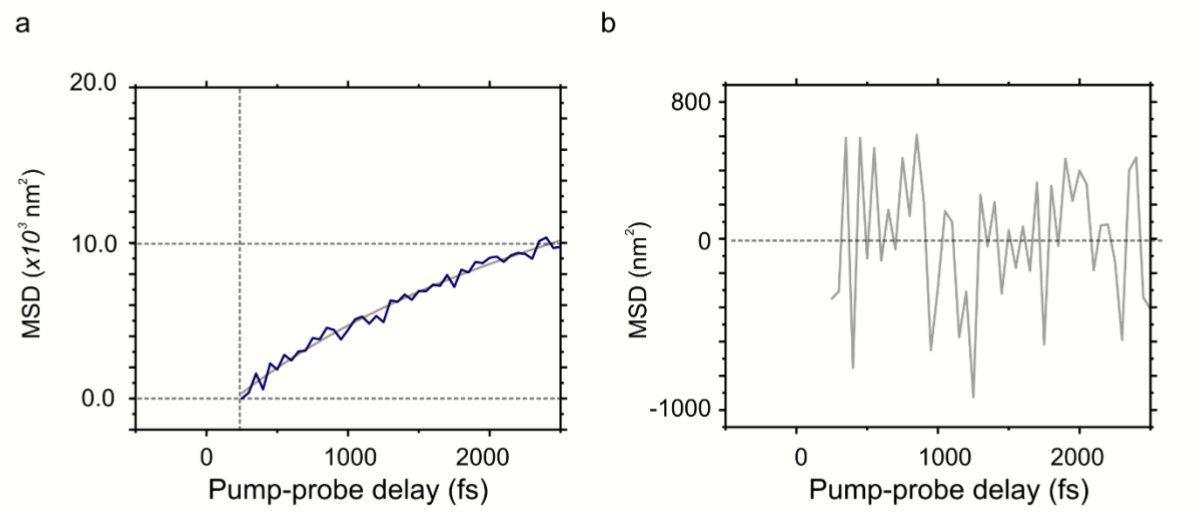

Figure S3: a. Typical fit of equation 3 of main text to MSD trace. **b.** Residual following fit of equation 3 to MSD trace.

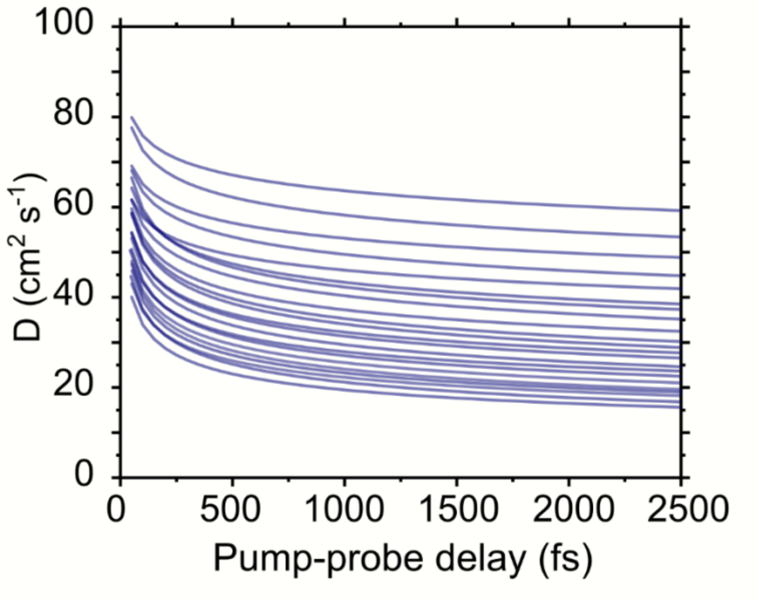

Figure S3i: Diffusivity, *D*, of 'blue' PDA plotted as a function of time for all traces in Figure 2 of main text.

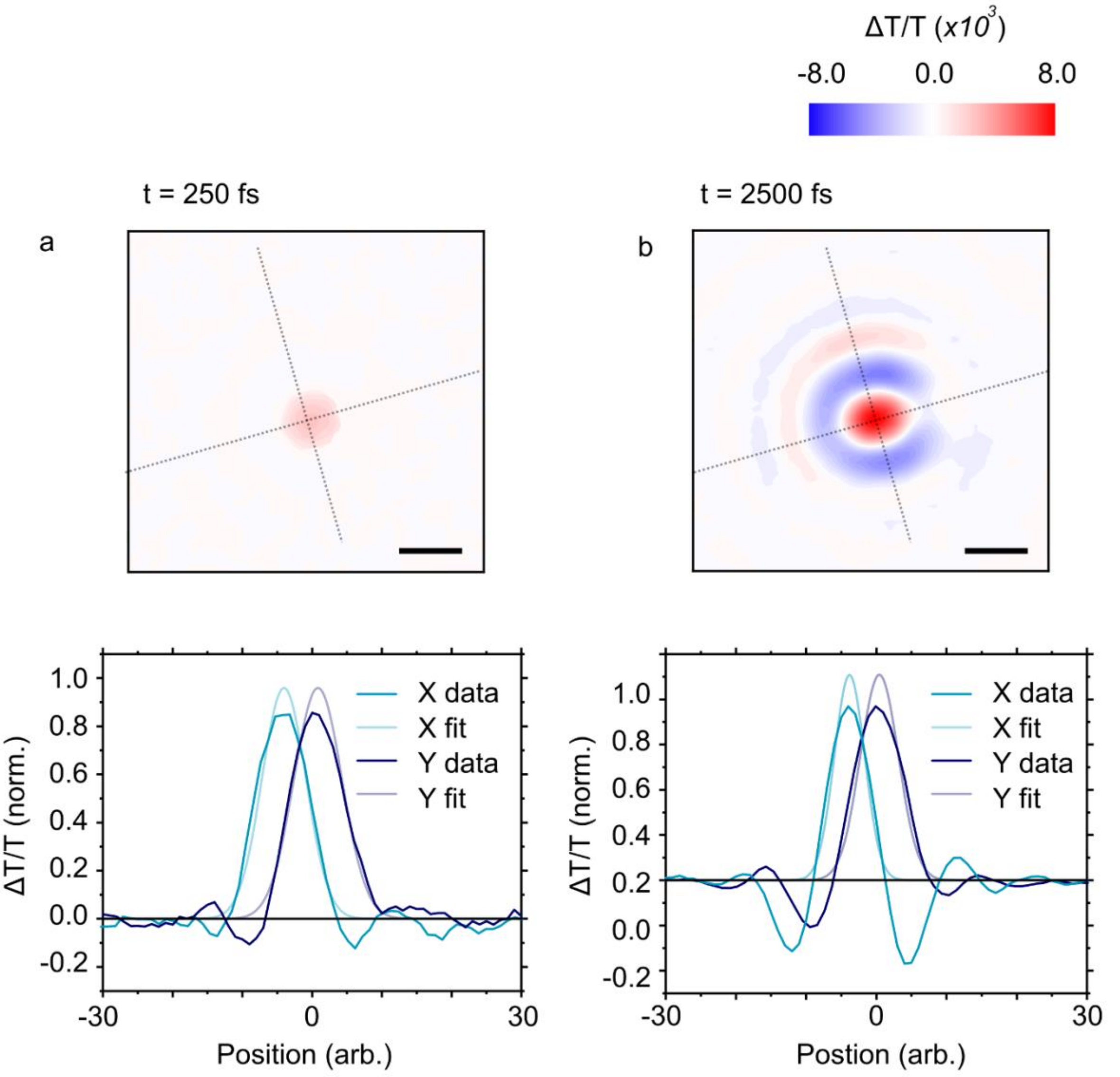

Figure S3ii: a-b. fs-TAM images for 'blue' PDA at 250 fs (a) and 2500 fs (b). The dashed lines show the directions of the principle axis of a 2D Gaussian fitted to the trace. Bottom shows typical line cuts of 2D Gaussian along the principle X (horizontal) and Y (vertical) axes, as well as the resulting fits.

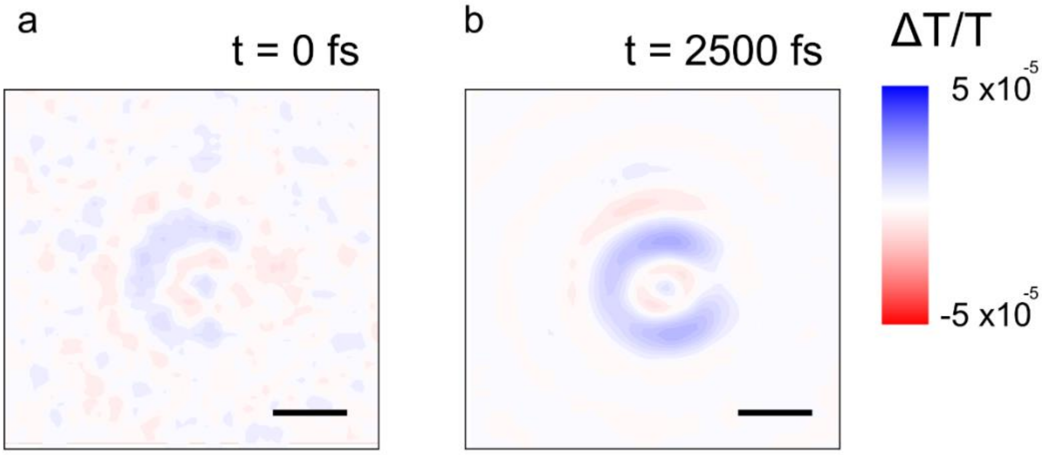

Figure S3iii: a-b. Residual following 2D Gaussian fit to images in Figure S3ii. The fit shows that the diffraction ring is reproduced faithfully. This implies that the deviation from a true two dimensional spatial profile in our measurements due to the diffraction ring has no effect on the retrieved spatial standard deviation. The slight asymmetry in our point spread function arises from imperfections in our imaging objective as noted in previous studies[3–5].

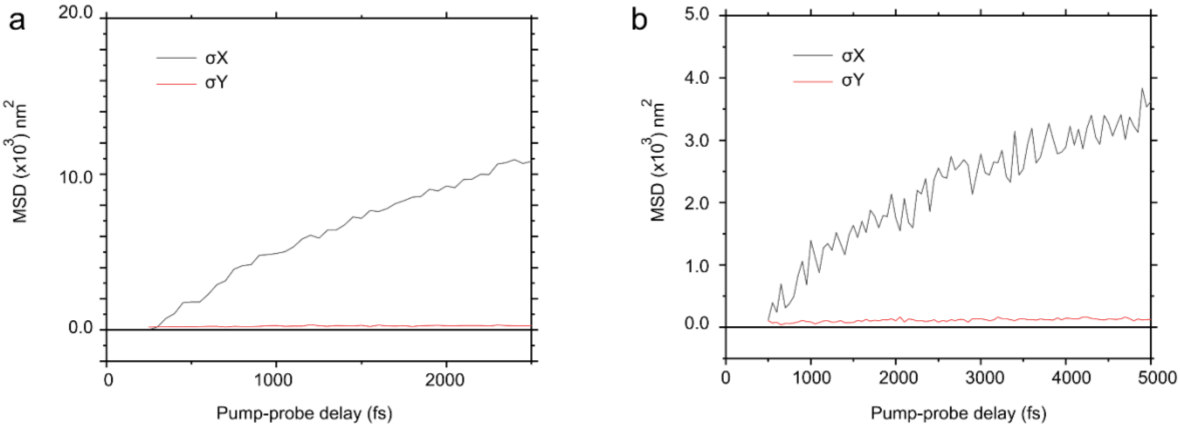

Figure S3iv: a-b. Mean-square displacement along principle axes for 'blue' and 'red' PDA. X is along wire direction and Y perpendicular to it.

S4: Pump fluence dependence of fs-TAM dynamics of ‘blue’ and ‘red’ PDA

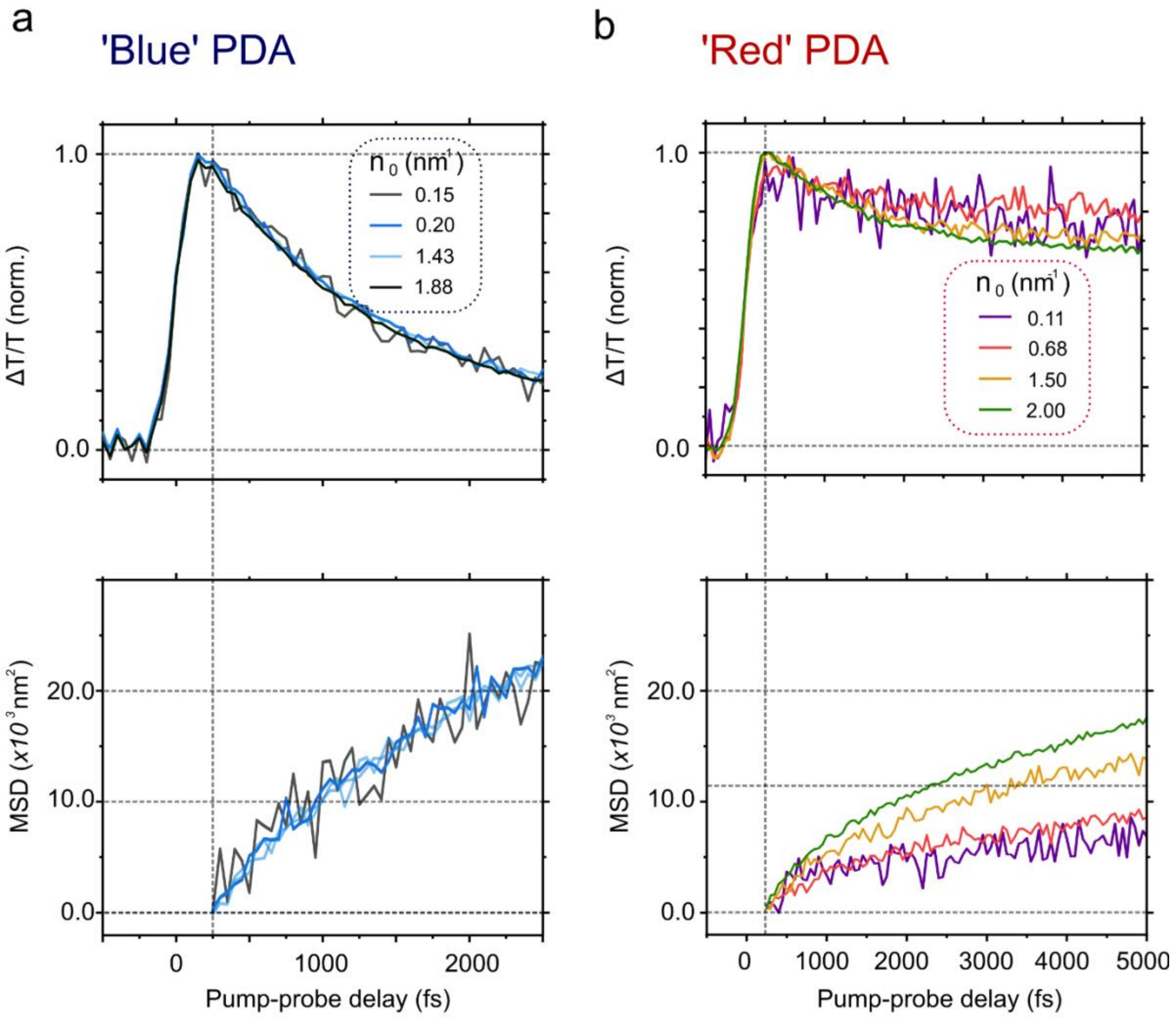

Figure S4: a-b. Pump-probe kinetics and MSD as a function of number of excitations per nm of polymer chain (n_0) for ‘blue’ and ‘red’ PDA. In the case of ‘blue’ PDA there is no dependence of the kinetics or MSD on the carrier density, whereas in ‘red’ PDA the decay quickens and diffusion coefficient increases at higher pump fluences. In this latter case exciton-exciton annihilation effects can be considered to be important.

To assess the role of defects fs-TAM measurements were repeated on thicker PDA crystals which have been shown to have lower energetic disorder[2] and less intrinsic defects. There is a negligible difference in the MSD traces between the two samples for both ‘red’ and ‘blue’ PDA as shown in Figure S4i (measurements performed under identical conditions). Fitting the traces gives diffusion coefficients of: blue D_{thick} = 27 ± 3 cm^2 s^{-1}, D_{thin} = 25 ± 2 cm^2 s^{-1}; red D_{thick} = 8 ± 0.8 cm^2 s^{-1}, D_{thin} = 7.6 ± 0.8 cm^2 s^{-1}. The difference in the electronic disorder between thick and thin crystals (~10%) is similar in magnitude to the difference in diffusion coefficient between these different materials. Tentatively this suggests that

disorder may be responsible for the observed differences, however further work is required to fully confirm this.

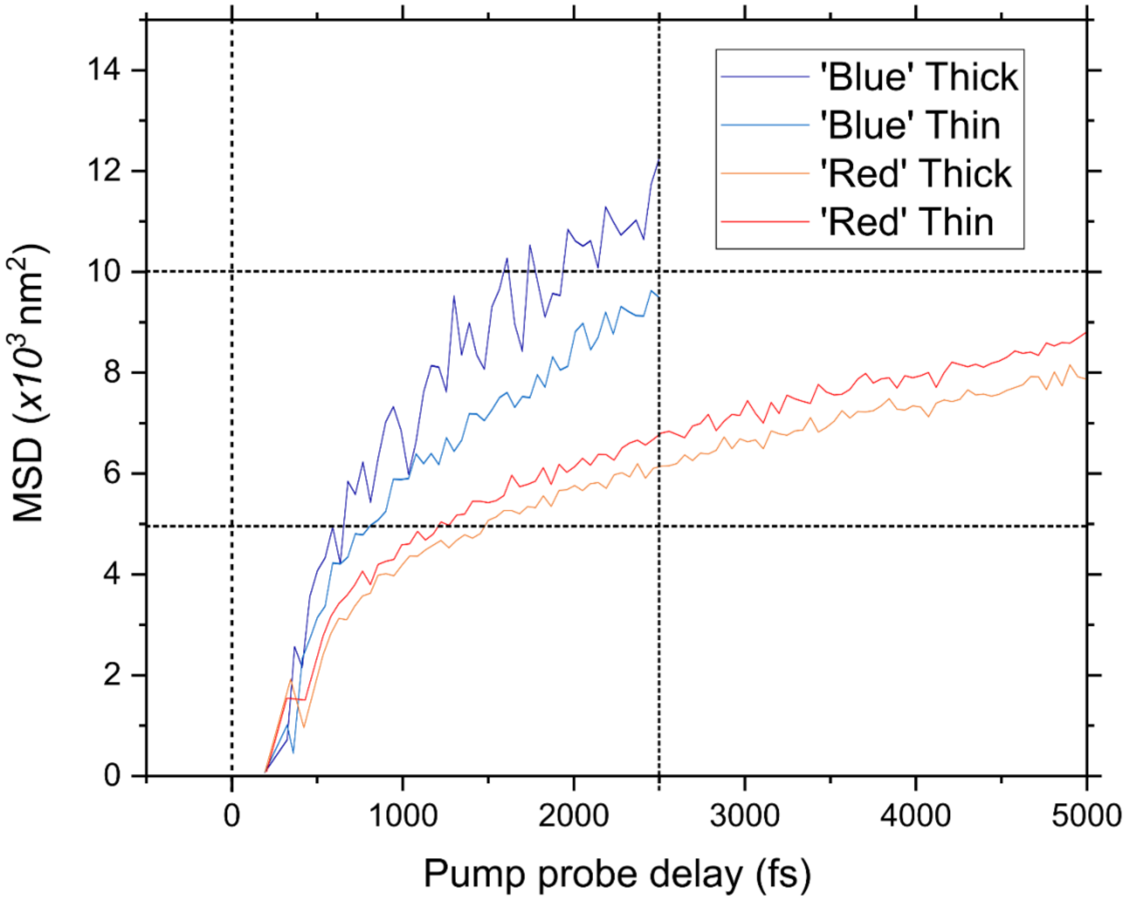

Figure S4i: MSD traces for ‘blue’ and ‘red’ PDA, ‘thick’ (0.1 mm) and ‘thin’ (0.0005 mm) crystals.

S5: Annihilation in ‘blue’ and ‘red’ PDA

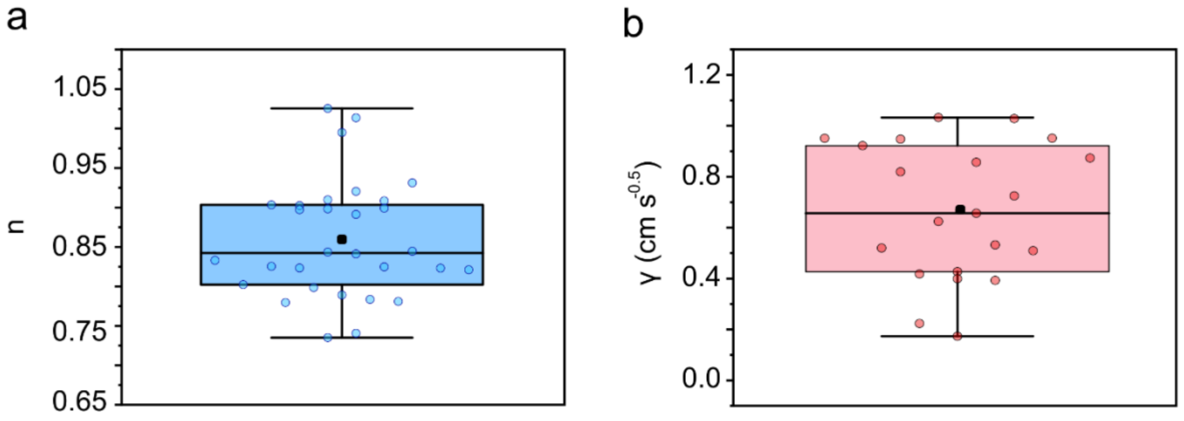

Figure S5: a. Box-scatter plot of n coefficient for ‘blue’ PDA. n consistently lies between 0.75 – 1.05 (mean value ~0.85) suggestive of sub-diffusive motion. **b.** Box-scatter plot of γ annihilation coefficient for ‘red’ PDA. There is a large variation in values between 0.1 – 1.1 cm s$^{-0.5}$

The separation between excitons is tabulated below for each of the fluences in our measurements. The calculation is performed as follows:

1. The excitation density, n, can then be calculated from $\frac{\varphi \times OD}{d}$ where φ is the photon flux, *OD* the optical density of film at the peak excitation wavelength and *d* is the film thickness.
2. The volume (V) of the chain is then calculated from the minimal separation between chains, representing the chain space.
3. n/V will give the excitations per chain (n_0), dividing this by the chain length ~2000-5000 nm will give the excitations per unit length (nm).

Excitations per cm^3	Chain volume	# chains per cm^3	# Excitations per chain	# Excitations per nm	Distance between excitations (nm)	# Excitations per repeat length
9.4E+16	7.85E-15	1.27E+14	7.38E+02	1.48E-01	6.77E+00	1.03E-01
1.3E+17	7.85E-15	1.27E+14	1.02E+02	2.04E-01	4.97E+00	1.43E-01
9.1E+17	7.85E-15	1.27E+14	7.15E+03	1.43E+00	7.00E-01	1.00E+00
1.2E+18	7.85E-15	1.27E+14	9.42E+03	1.88E+00	5.31E-01	1.32E+00

Excitations per cm^3	Chain volume	# chains per cm^3	# Excitations per chain	# Excitations per nm	Distance between excitations (nm)	# Excitations per repeat length
7.1E+16	7.85E-15	1.27E+14	5.58E+02	1.12E-01	8.97E+00	7.81E-01
4.3E+17	7.85E-15	1.27E+14	3.38E+02	6.75E-01	1.48E+00	4.73E-01
9.2E+17	7.85E-15	1.27E+14	7.23E+03	1.45E+00	6.92E-01	1.00E+00
1.3E+18	7.85E-15	1.27E+14	1.02E+04	2.04E+00	4.90E-01	1.43E+00

Table S1: Table summarising excitation densities and corresponding distance between excitons in ‘blue’ (top) and ‘red’ (bottom) PDA respectively.

The closest that excitons approach in the fluence range used in our measurements is ~0.5 nm. Based on a repeat unit length of ~0.7 nm, this suggests that even at the highest fluences there is only 1.3 – 1.4 excitons per repeat unit. Consequently, it can be assumed that annihilation *via* impact ionization and Auger scattering are minimal.

Although Figure S5 captures the spread in annihilation and diffusion coefficients at a single fluence, performing a fluence series at multiple sample locations allows us to further quantify the heterogeneity as shown in Figure S5i.

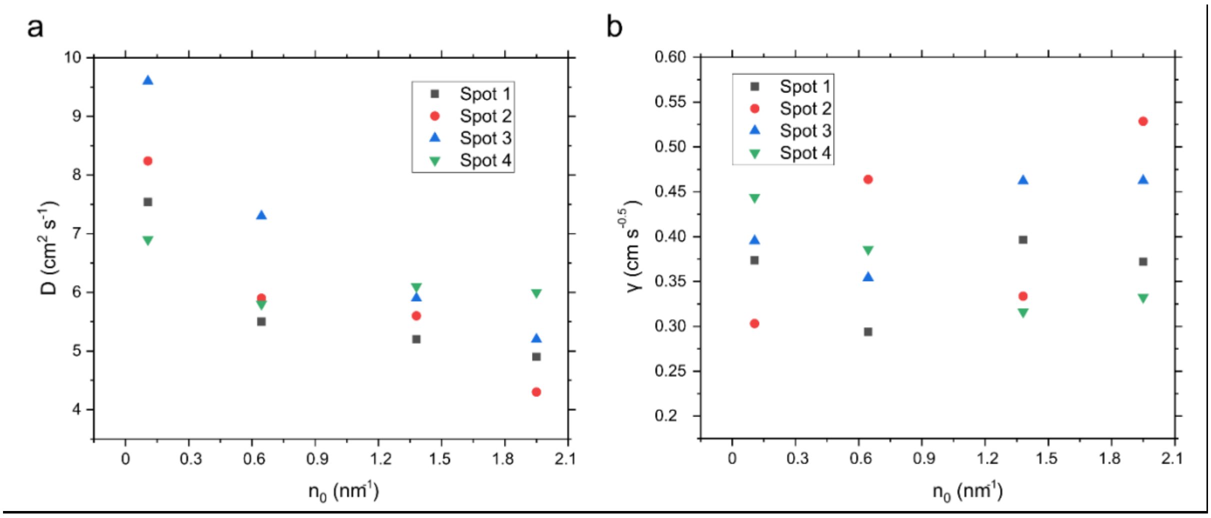

Figure S5i: a-b. Distribution of diffusion coefficient D and annihilation coefficient γ at 4 different sample locations for 'red' PDA.

For γ an overall: standard deviation = 0.36; mean = 0.39 cm $s^{-0.5}$; range = 0.21 cm $s^{-0.5}$ whereas for D an overall: standard deviation = 1.35; mean = 6.2 cm^2 s^{-1}; range = 4.3 cm^2 s^{-1}. As discussed in the main text fixing D was found to produce an unsatisfactory fit. However, leaving D and γ free as fitting parameters produces satisfactory fits as shown in Figure S5ii. The values of D and γ reported in the main text are at the lowest fluence used here (~0.11 excitations per nm of polymer chain).

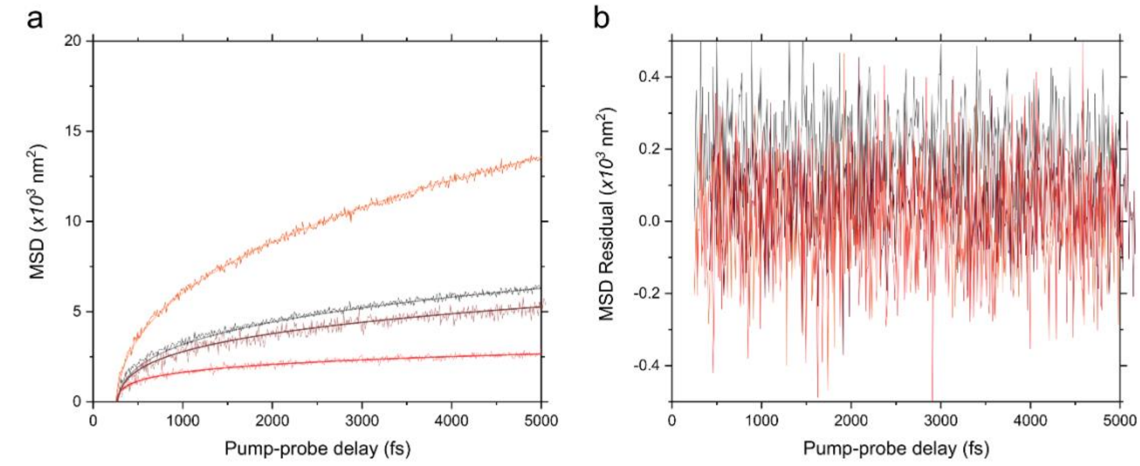

Figure S5ii: a. MSD traces at four different carrier densities for 'red' PDA. Solid line shows fit to data. **b.** Residual following subtraction of fit in **a**.

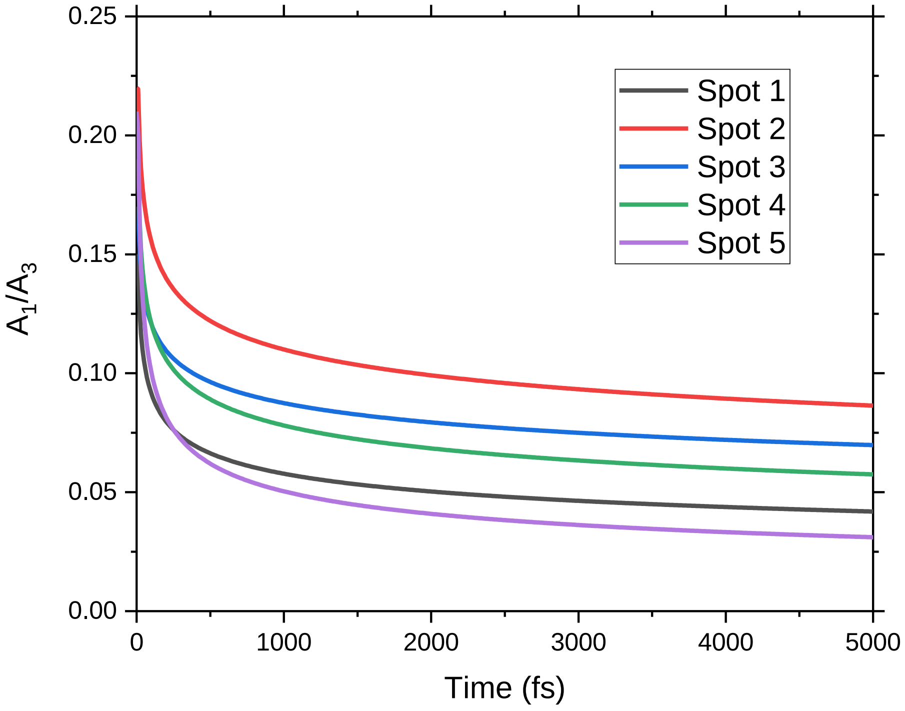

Figure S5iii: Ratio of first (A_1) and third (A_3) terms of equation 1 in main text. The diffusion terms is typically ~5 – 10 times larger than the annihilation term, suggesting the former process dominates.

S6: Calculation of the exciton properties of PDA

We calculate the exciton properties of PDA by employing the highly accurate Green's-function-based *GW*-BSE approximation[6–8], as implemented in the YAMBO code[9]. Due to the large computational cost associated with these calculations, especially when combining them with phonon effects (see section S7), we study a model PDA system, which is based on a realistic PDA structure deposited in the Cambridge Crystallographic Database (identifier: DEMYID). In the model system, the side-chains have been substituted by hydrogen atoms, hence limiting it to the conjugated structure along which exciton transport occurs.

For the energy self-consistent *GW* calculation, the parameters that converge the fundamental gap are 40 Kohn-Sham states, 40 states for the polarisation function calculation, and a 3 Ry cut-off for the dielectric matrix. The solution of the Bethe-Salpeter equation (BSE) yields the exciton energy of the $1^1B_u^+$ exciton state, which is converged for 4 occupied and 4 unoccupied bands, and setting the exchange term to 40 Ry. To avoid divergences that appear in the Coulomb term at long distances (small q), we utilise the random integration method with 10^5random q-points in the first Brillouin zone, setting the cut-off for the real space components of the Coulomb interaction to 20 Ry.

We estimate the excitonic J coupling between neighboring monomers of diacetylene by computing the width of the $1^1B_u^+$ exciton band, which is equal to $4J$ within a tight-binding picture, therefore

$$J = \frac{E(X) - E(\Gamma)}{4},$$

where E the exciton energy at the points $q(\Gamma) = (0,0,0)$ (the zone-center), and $q(X) = (\frac{2\pi}{\alpha}, 0,0)$ the zone-edge in the x-direction, along which the PDA chains lie. To calculate $E(X)$, we solve the BSE at the band-edge, as implemented in YAMBO. We thus obtain $J = 0.54$ eV.

Finally, it is worth highlighting that the above supercell calculation allows us to estimate the exciton group velocity as

$$v_g = \frac{1}{\hbar} \cdot \frac{E(X) - E(\Gamma)}{q(X) - q(\Gamma)} = 3 \cdot 10^5 \ \text{m/s},$$

which we use for the calculation of the exciton coherence length in section S7.

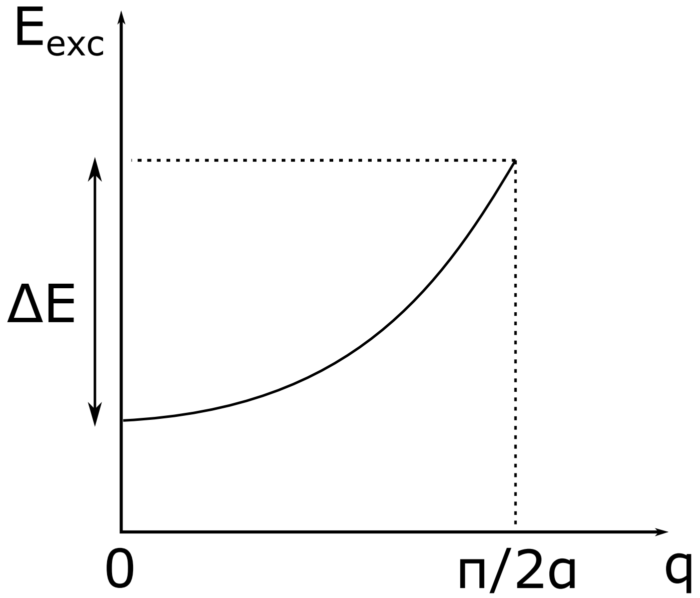

Figure S6: Visualization of the exciton dispersion in reciprocal space.

S7: Theoretical estimation of exciton-phonon effects in PDA

Exciton-phonon coupling and reorganization energy

In this section, we study the model PDA structure which is described in section S6. Following a geometry optimisation within density functional theory using the Quantum Espresso software package[10] and the PBE functional, we perform a phonon calculation using finite differences[11].

We then calculate the exciton-phonon coupling elements for each mode k as

$$V_{ep,k} = \frac{E_{exc}(+\delta u) - E_{exc}(-\delta u)}{2\delta u},$$

where δu a small displacement of the equilibrium structure. Hence for every vibrational mode, two GW-BSE calculations are necessary in order to obtain $V_{ep,k}$. The Huang-Rhys factors are obtained as[12]

$$S_k = \frac{1}{N} \cdot \left(\frac{V_{ep,k}}{\hbar\omega_k}\right)^2,$$

with N being the exciton coherence length, i.e. the number of diacetylene monomers that the exciton can travel over coherently, before being scattered by a phonon

$$N = \frac{v_g \cdot \tau_{scatter}}{L_{DA}}.$$

Here v_g the exciton group velocity computed in section S6, L_{DA} the length of a single monomer of diacetylene, and $\tau_{scatter}$ the characteristic scattering timescale. The calculation of $V_{ep,k}$ reveals that the double-bond stretching motion has the steepest excited state surface, *i.e.* the largest $V_{ep,k}/\hbar\omega_k$ value, and will dominate the scattering. We thus approximate $\tau_{scatter} = 23$ fs, which is the period of this mode, and we find $N = 15$ monomers. The values of $V_{ep,k}$ and S_k are summarized alongside the frequencies ω_k of the respective vibrations in Table S2. From these, the reorganization energy is approximated as[13]

$$\lambda = 2\sum_k \hbar\omega_k S_k = 0.43 \text{ eV}.$$

**Mode frequency $\boldsymbol{\omega_k}$ ($\mathbf{cm^{-1}}$)**	**$\boldsymbol{V_{ep,k}}$ (meV)**	**$\boldsymbol{S_k}$**
389	2	0
398	7	0
427	-2	0
435	1	0
823	2	0
924	-86	0.09

928	-76	0.07
1228	1	0
1255	192	0.23
1386	4	0
1488	-310	0.43
2115	-383	0.33
3085	9	0
3093	-1	0

Table S2. Exciton-phonon coupling elements and Huang-Rhys factors for the $1^1B_u^+$ exciton state of the model PDA system described in section S6.

We note that the coherence length of 15 monomers is typically higher than other polymer systems. For example, in MEH-PPV the coherence length at 300 K is 6 [14], it is 3 in P3HT [15] and 5 [16] in PDI aggregates. However, in previous studies the coherence length of PDA polymers has been reported to extend over the entire length of the polymer chain albeit at 4 K[17], hence the large coherence length calculated is not unreasonable. Indeed, in highly ordered aggregate systems with similar values of *J*-coupling [18] large coherence lengths have been reported at up to 20 molecules.[19] Simulations by Spano *et al.*[19] also indicate an exciton coherence length of 350 Angstrom in planar PDA, which is confirmed by experimental measurements using the Franz-Keldysh effect[20]. This corresponds to approximately 70 repeat units for the exciton coherence length. Therefore, our value of 15 is between the two extremes seems to be a reasonable estimate. We hence suggest the large coherence length in PDA reported to be reasonable and a consequence of the low disorder[21] and strong inter-monomer interactions[2].

From Table S2 we see that the carbon-carbon double-bond stretch (1488 cm^{-1}) and the triple-bond stretch (2115 cm^{-1}) are the vibrational modes most strongly coupled to the exciton. This can intuitively be understood from the fact that these motions allow the polymer chains to transiently explore configurations closer to the limit of a structure without Peierls distortion and hence to the metallic limit [22]. Another way of understanding this is that compared to other vibrational motions, these phonon modes lead to strong changes to the excitonic wavefunction once they are displaced, as visualized in Figure S6 for the case of the double-bond stretch. The electronic density of the exciton wavefunction is plotted in red for a hole localized at the blue dot in each case. We compare the case of the undistorted geometry, to that of displacing the structure along the weakly coupled lowest frequency mode, and to that of displacing along the double-bond stretching motion. The latter evidently leads to a significant change in the excitonic wavefunction, altering the bonding pattern of the electronic density. For visualization purposes, here we have exaggerated the displacement of the phonons compared to its values at room temperature.

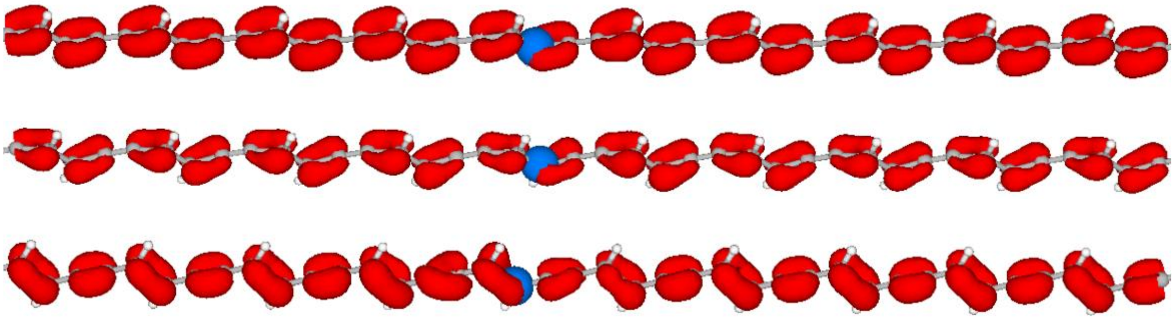

Figure S7: Visualization of exciton wavefunctions. The electronic density is given in red for a hole localized at the position in blue for **a.** the undistorted geometry **b.** displacement along the 389 cm^{-1} phonon, **c.** displacement along the 1488 cm^{-1} phonon (double-bond stretch).

Phonon-induced renormalization of excitonic observables

Observable quantities often undergo a renormalization at a given finite temperature due to the presence of phonons. In particular, when accounting for phonons, the value of some observable A at temperature T is[23]:

$$A(T) = A(\boldsymbol{u} = 0) + \sum_{\boldsymbol{q},k} \frac{1}{2\omega_{\boldsymbol{q}k}} \cdot \frac{\partial^2 A}{\partial u_{\boldsymbol{q}k}^2} \left[\frac{1}{2} + n_B\left(\omega_{\boldsymbol{q}k}, T\right)\right] + O(u^4),$$

where $\omega_{\boldsymbol{q}k}, u_{\boldsymbol{q}k}$ the frequency and displacement of phonon mode k with momentum $\boldsymbol{q}$, and n_B the Bose-Einstein distribution. This quadratic approximation is accurate up to third order in u, and we employ it in order to obtain the exciton energy, the excitonic coupling J, and the magnitude of the transition dipole moment $|\boldsymbol{\mu}|$ at 300 K. The obtained value becomes increasingly accurate with the inclusion of more $\boldsymbol{q}$-points. For the coupling to excitons, phonons at the Γ ($\boldsymbol{q} = (0,0,0)$) and X ($\boldsymbol{q} = (\frac{2\pi}{\alpha}, 0,0)$) points dominate the interaction[24], and we hence restrict the calculation to these two points in order to keep the computational cost at a minimum (the evaluation of the second derivative for each mode requires two GW-BSE calculations, in the $+\delta u$ and $-\delta u$ directions).

The computed static exciton energy in the planar PDA is found to be 1.88 eV, with a renormalization of $+14$ meV due to phonons at 300 K. A strong static J coupling of 0.54 eV is found (see section S6), with only a small renormalization of -5 meV. Finally, the absolute magnitude of the transition dipole moment is found to have a value of 1.82 a.u. at the ground state geometry, and accounting for phonons is found to lead to a significant renormalization of its value by -0.52 a.u.. While the quadratic approximation can be less accurate compared to a Monte Carlo sampling of the phonon-induced renormalization of the observable A [25], it has the advantage that it provides mode-resolved information. Therefore, in Table S3 we report the specific contribution of the individual modes to the total renormalization reported here. For the transition dipole moment, which is the only quantity among the

three studied ones with a non-negligible renormalization, the modes with frequencies 1488 cm^{-1} and 2115 cm^{-1} are found to dominate, as was also the case for exciton-phonon coupling.

**Mode frequency** $\boldsymbol{\omega_k}$ $(\mathbf{cm^{-1}})$	$\boldsymbol{\delta E_{exc,k}}$ $(\mathbf{meV})$	$\boldsymbol{\delta J_k}$ $(\mathbf{meV})$	$\boldsymbol{\delta\lvert\mu\rvert}(\mathbf{a.u.})$
389	0.4	-0.7	0.003
398	1.1	1.1	0.024
427	2.3	-2.3	-0.003
435	1.4	-0.1	-0.002
823	1.9	0.1	0.004
924	1	-1.1	-0.087
928	1.2	-1.3	-0.077
1228	0.5	-0.3	0.001
1255	1.3	-0.8	0.184
1386	-0.3	0.1	0.004
1488	0.4	-0.1	-0.265
2115	2.7	1	-0.318
3085	0.6	-0.2	0.008
3093	0	-0.1	-0.001

Table S3. Renormalization of the exciton energy, excitonic coupling and absolute magnitude of the transition dipole moment due to individual phonon modes at 300 K.

References

(1) Spagnoli, S.; Berrehar, J.; Fave, J. L.; Schott, M. Temperature Dependence of Spectroscopic Properties of Isolated Polydiacetylene Chains Strained by Their Monomer Single Crystal Matrix. *Chem. Phys.* **2007**, *333* (2), 254–264..

(2) Pandya, R.; Gu, Q.; Cheminal, A.; Chen, R. Y. S.; Booker, E. P.; Soucek, R.; Schott, M.; Legrand, L.; Mathevet, F.; Greenham, N. C.; et al. Optical Projection and Spatial Separation of Spin Entangled Triplet-Pairs from the S1 ($2^1A_g^-$) State of Pi-Conjugated Systems. *Chem.* **2020**, *6*, (10), 2826-2851

(3) Schnedermann, C.; Sung, J.; Pandya, R.; Verma, S. D.; Chen, R. Y. S.; Gauriot, N.; Bretscher, H. M.; Kukura, P.; Rao, A. Ultrafast Tracking of Exciton and Charge Carrier Transport in Optoelectronic Materials on the Nanometer Scale. *J. Phys. Chem. Lett.* **2019**, *10* (21), 6727–6733.

(4) Sung, J.; Schnedermann, C.; Ni, L.; Sadhanala, A.; Chen, R. Y. S.; Cho, C.; Priest, L.; Lim, J. M.; Kim, H.-K.; Monserrat, B.; et al. Long-Range Ballistic Propagation of Carriers in Methylammonium Lead Iodide Perovskite Thin Films. *Nat. Phys.* **2019**, *16*, 171–176.

(5) Pandya, R.; Chen, R. Y. S.; Gu, Q.; Gorman, J.; Auras, F.; Sung, J.; Friend, R.; Kukura, P.; Schnedermann, C.; Rao, A. Femtosecond Transient Absorption Microscopy of Singlet Exciton Motion in Side-Chain Engineered Perylene-Diimide Thin Films. *J. Phys. Chem. A* **2020**, *124* (13), 2721–2730.

(6) Hedin, L. New Method for Calculating the One-Particle Green’s Function with Application to the Electron-Gas Problem. *Phys. Rev.* **1965**. *139* A796

(7) Rohlfing, M.; Louie, S. G. Electron-Hole Excitations in Semiconductors and Insulators. *Phys. Rev. Lett.* **1998**. *11*, (81)

(8) Marini, A.; Hogan, C.; Grüning, M.; Varsano, D. Yambo: An Ab Initio Tool for Excited State Calculations. *Comput. Phys. Commun.* **2009**, *180*, (8), 1392-1403

(9) Sangalli, D.; Ferretti, A.; Miranda, H.; Attaccalite, C.; Marri, I.; Cannuccia, E.; Melo, P.; Marsili, M.; Paleari, F.; Marrazzo, A.; et al. Many-Body Perturbation Theory Calculations Using the Yambo Code. *J. Phys. Condens. Matter* **2019**, *31* (32).

(10) Giannozzi, P.; Baroni, S.; Bonini, N.; Calandra, M.; Car, R.; Cavazzoni, C.; Ceresoli, D.; Chiarotti, G. L.; Cococcioni, M.; Dabo, I.; et al. QUANTUM ESPRESSO: A Modular and Open-Source Software Project for Quantum Simulations of Materials. *J. Phys. Condens. Matter* **2009**, *21* (39) 5502

(11) Kunc, K.; Martin, R. M. Ab Initio Force Constants of Gaas: A New Approach to Calculation of Phonons and Dielectric Properties. *Phys. Rev. Lett.* **1982**. *48*, 406

(12) Spano, F. C. Optical Microcavities Enhance the Exciton Coherence Length and Eliminate Vibronic Coupling in J-Aggregates. *J. Chem. Phys.* **2015**., *142*, 14807

(13) Aragó, J.; Troisi, A. Regimes of Exciton Transport in Molecular Crystals in the Presence of Dynamic Disorder. *Adv. Funct. Mater.* **2016**, *26,* (14), 2316-2325

(14) Yamagata, H.; Hestand, N. J.; Spano, F. C.; Köhler, A.; Scharsich, C.; Hoffmann, S. T.; Bässler, H. The Red-Phase of Poly[2-Methoxy-5-(2-Ethylhexyloxy)- 1,4-Phenylenevinylene] (MEH-PPV): A Disordered HJ-Aggregate. *J. Chem. Phys.* **2013**. 139, 114903

(15) Spano, F. C.; Clark, J.; Silva, C.; Friend, R. H. Determining Exciton Coherence from the Photoluminescence Spectral Line Shape in Poly(3-Hexylthiophene) Thin Films. *J. Chem.*

Phys. **2009**. 130, 074904

(16) Sung, J.; Kim, P.; Fimmel, B.; Würthner, F.; Kim, D. Direct Observation of Ultrafast Coherent Exciton Dynamics in Helical π-Stacks of Self-Assembled Perylene Bisimides. *Nat. Commun.* **2015**, *6* (1), 8646.

(17) Dubin, F.; Melet, R.; Barisien, T.; Grousson, R.; Legrand, L.; Schott, M.; Voliotis, V. Macroscopic Coherence of a Single Exciton State in an Organic Quantum Wire. *Nat. Phys.* **2006**, *2* (1), 32.

(18) Scholes, G. D. Limits of Exciton Delocalization in Molecular Aggregates. *Faraday Discuss.* **2019**.

(19) Spano, F. C.; Yamagata, H. Vibronic Coupling in J-Aggregates and beyond: A Direct Means of Determining the Exciton Coherence Length from the Photoluminescence Spectrum. *J. Phys. Chem. B* **2011**. *115*, (18), 5133–5143

(20) Horvath, A.; Weiser, G.; Lapersonne-Meyer, C.; Schott, M.; Spagnoli, S. Wannier Excitons and Franz-Keldysh Effect of Polydiacetylene Chains Diluted in Their Single Crystal Monomer Matrix. *Phys. Rev. B - Condens. Matter Mater. Phys.* **1996**, *53*, 13507–13514.

(21) Schott, M. The Colors of Polydiacetylenes: A Commentary. *J. Phys. Chem. B* **2006**, *110* (32), 15864–15868.

(22) Kivelson, S.; Heeger, A. J. First-Order Transition to a Metallic State in Polyacetylene: A Strong-Coupling Polaronic Metal. *Phys. Rev. Lett.* **1985**, *55* (3), 308–311.

(23) Monserrat, B. Electron – Phonon Coupling from Finite Differences. *J. Phys. Condens. Matter* **2018**, *30*.

(24) Alvertis, A. M.; Pandya, R.; Muscarella, L. A.; Sawhney, N.; Nguyen, M.; Ehrler, B.; Rao, A.; Friend, R. H.; Chin, A. W.; Monserrat, B. Impact of Exciton Delocalization on Exciton-Vibration Interactions in Organic Semiconductors. *Phys. Rev. B Phys.* **2020**, *102*, 081122(R)..

(25) Monserrat, B. Vibrational Averages along Thermal Lines. *Phys. Rev. B* **2016**, *93* (1), 1–10.
